# Supplementary material for: Rhodamine6G and Hœchst33342 narrow BmrA conformational spectrum for a more efficient use of ATP
Source: Nat Commun. 2025 Feb 18;16:1745. doi: 10.1038/s41467-025-56849-z (PMC11836358; doi:10.1038/s41467-025-56849-z)
Supplement: Supplementary file 1 — Supplementary Information [file 41467_2025_56849_MOESM1_ESM.pdf]

Rhodamine6G and Hœchst33342 narrow BmrA conformational spectrum for a more efficient use of ATP

Supplementary Information

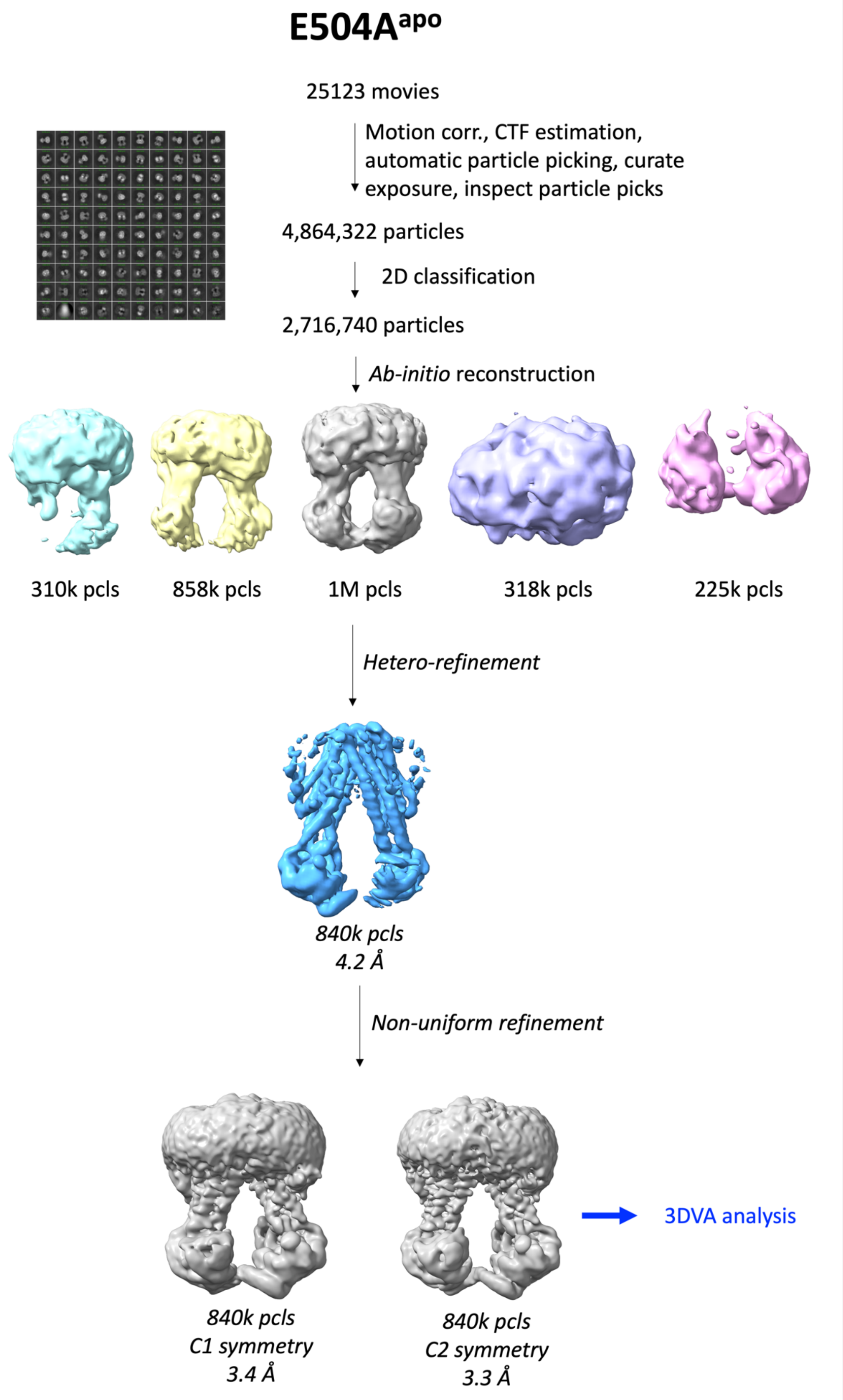

Supp-Figure 1: Data processing scheme for BmrA E504A^{apo}. Particles (pcls) are listed for each step and class, and resolution at FSC$^{=0.143}$ are listed for the latest stages of refinement.

Many routes were explored to reach high resolution reconstructions, only the final one is displayed.

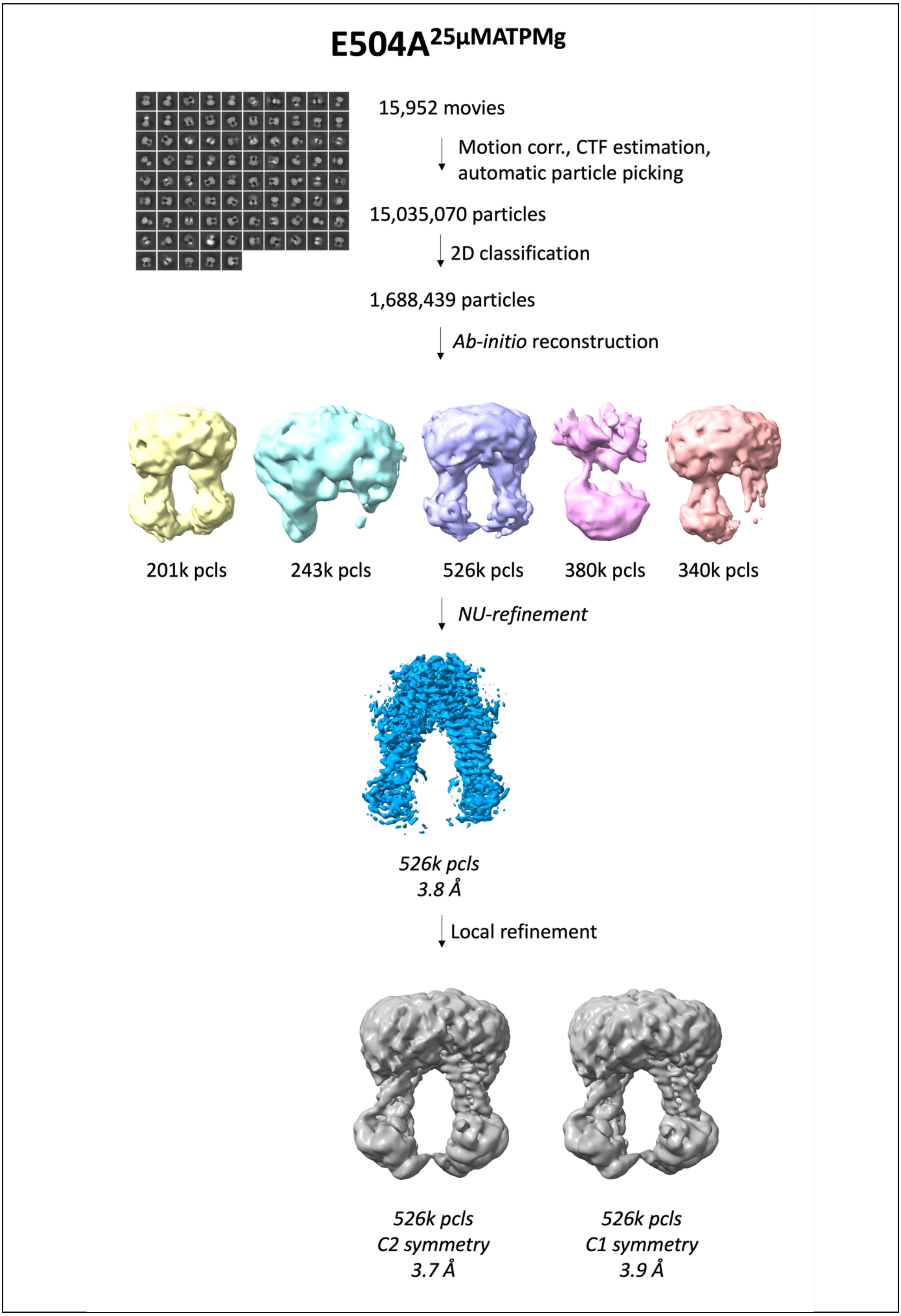
E504A25µMATPMg
15,952 movies
Motion corr., CTF estimation, automatic particle picking
15,035,070 particles
2D classification
1,688,439 particles
Ab-initio reconstruction
201k pcls
243k pcls
526k pcls
380k pcls
340k pcls
NU-refinement
526k pcls
3.8 Å
Local refinement
526k pcls
C2 symmetry
3.7 Å
526k pcls
C1 symmetry
3.9 Å

Supp-Figure 2: Data processing scheme for BmrA E504A$^{25\mu MATPMg}$. Particles (pcls) are listed for each step and class, and resolution at FSC$^{=0.143}$ are listed for the latest stages of refinement. Many routes were explored to reach high resolution reconstructions, only the final one is displayed.

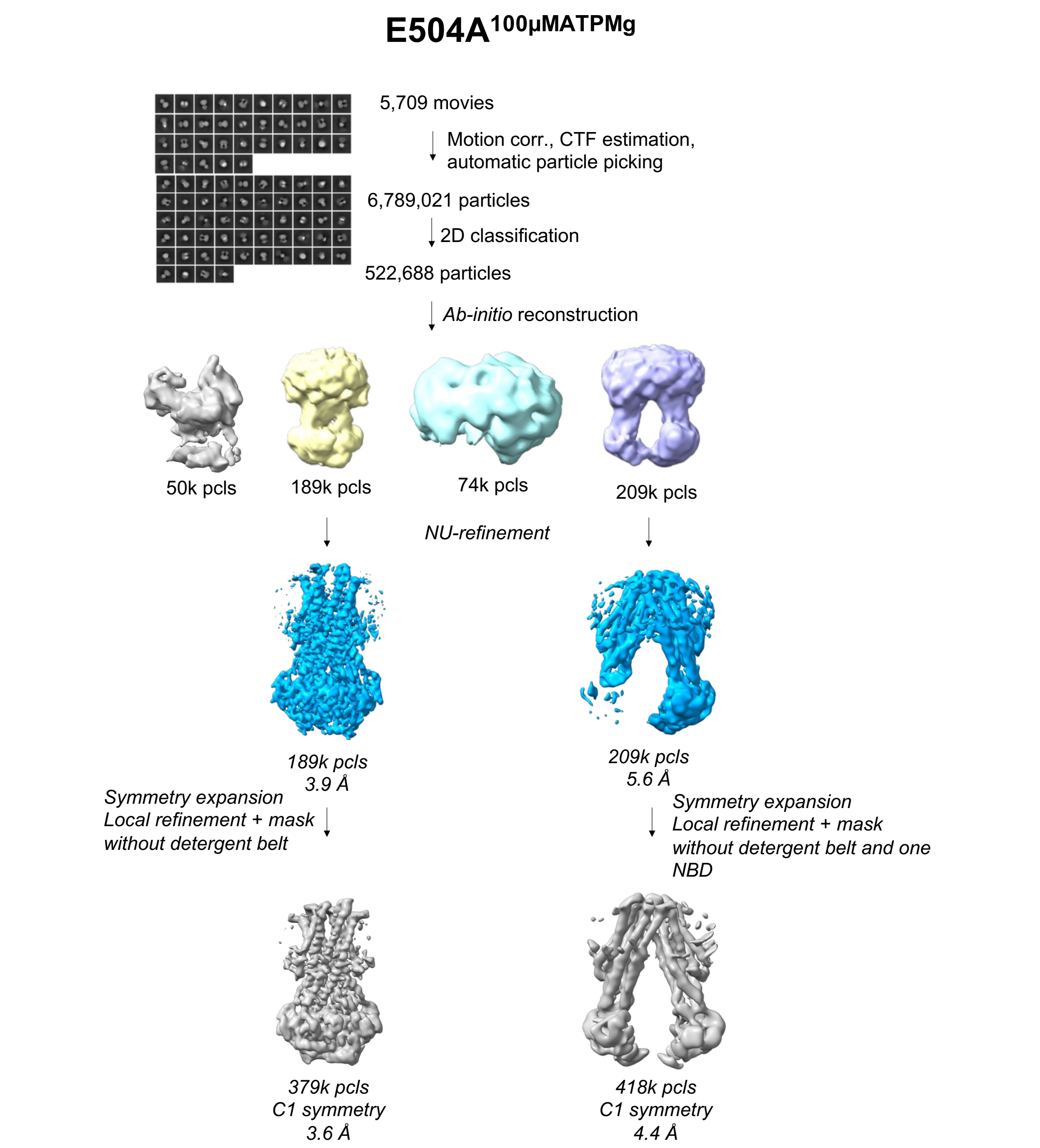

Supp-Figure 3: Data processing scheme for BmrA E504A$^{100\mu MATPMg}$. Particles (pcls) are listed for each step and class, and resolution at FSC$^{=0.143}$ are listed for the latest stages of refinement. Many routes were explored to reach high resolution reconstructions, only the final one is displayed.

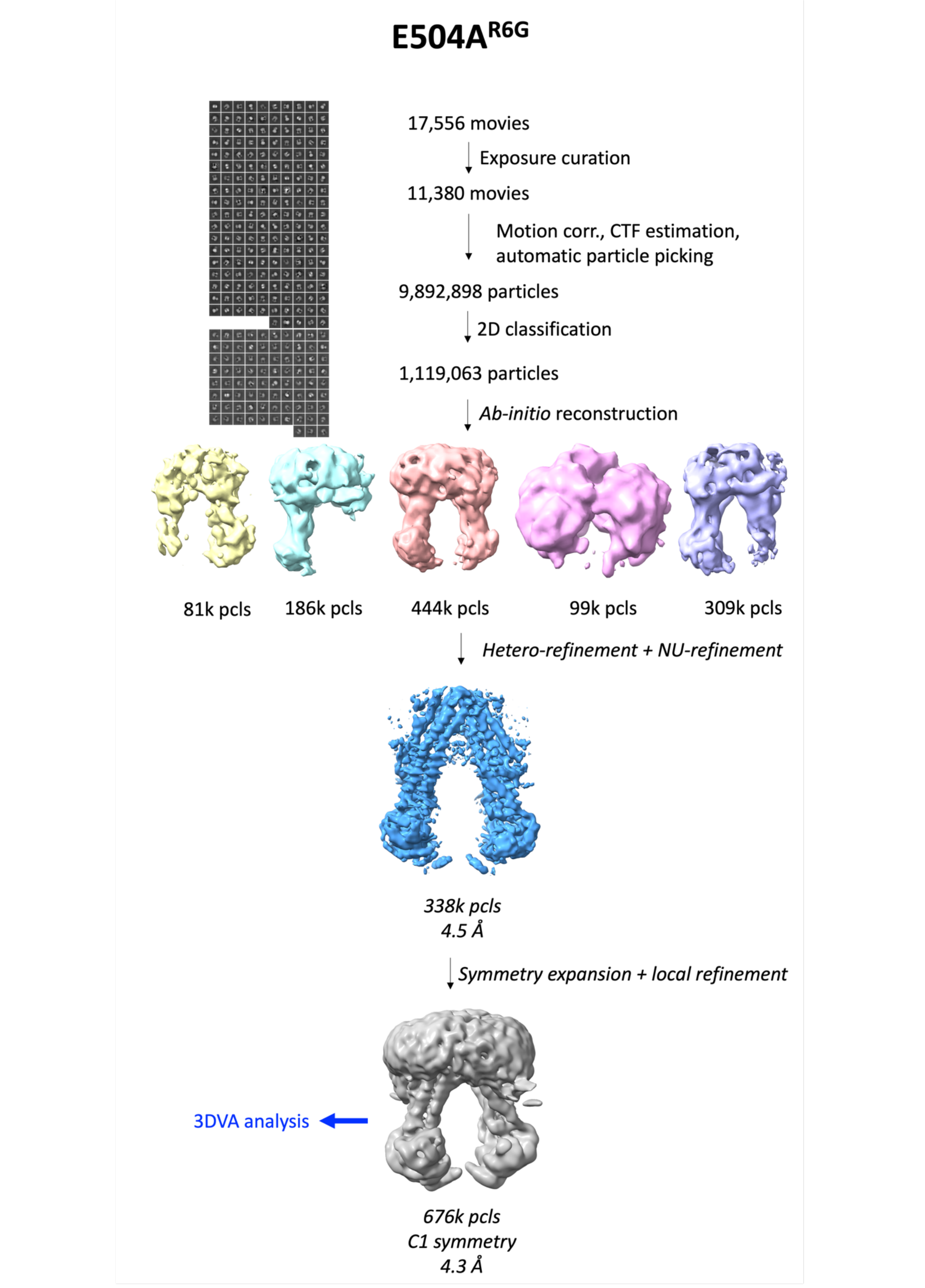

Supp-Figure 4: Data processing scheme for BmrA E504A^{R6G}. Particles (pcls) are listed for each step and class, and resolution at FSC$^{=0.143}$ are listed for the latest stages of refinement. Many routes were explored to reach high resolution reconstructions, only the final one is displayed.

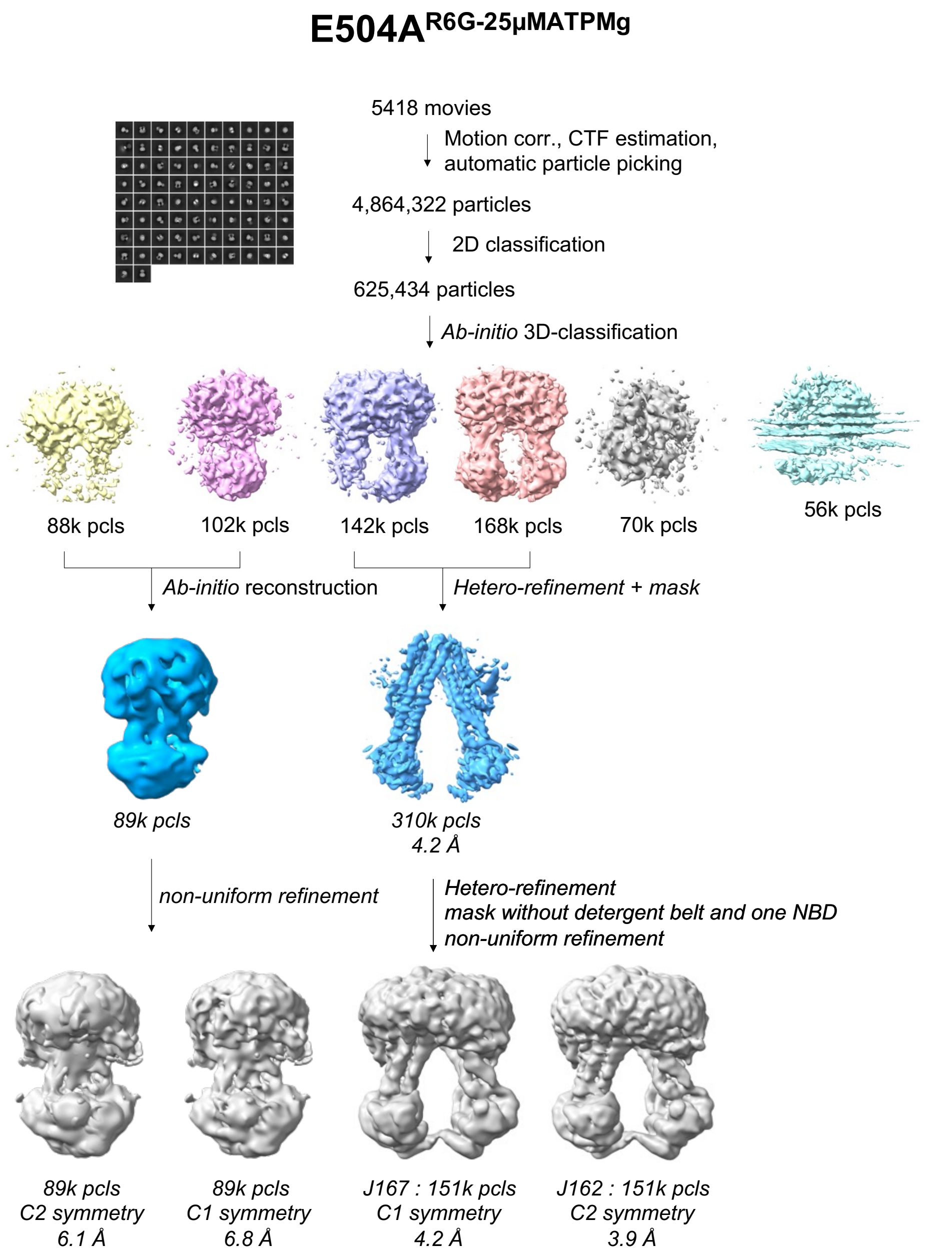

Supp-Figure 5: Data processing scheme for BmrA E504A$^{R6G\text{-}25\mu MATPMg}$. Particles (pcls) are listed for each step and class, and resolution at FSC$^{=0.143}$ are listed for the latest stages of refinement. Many routes were explored to reach high resolution reconstructions, only the final one is displayed.

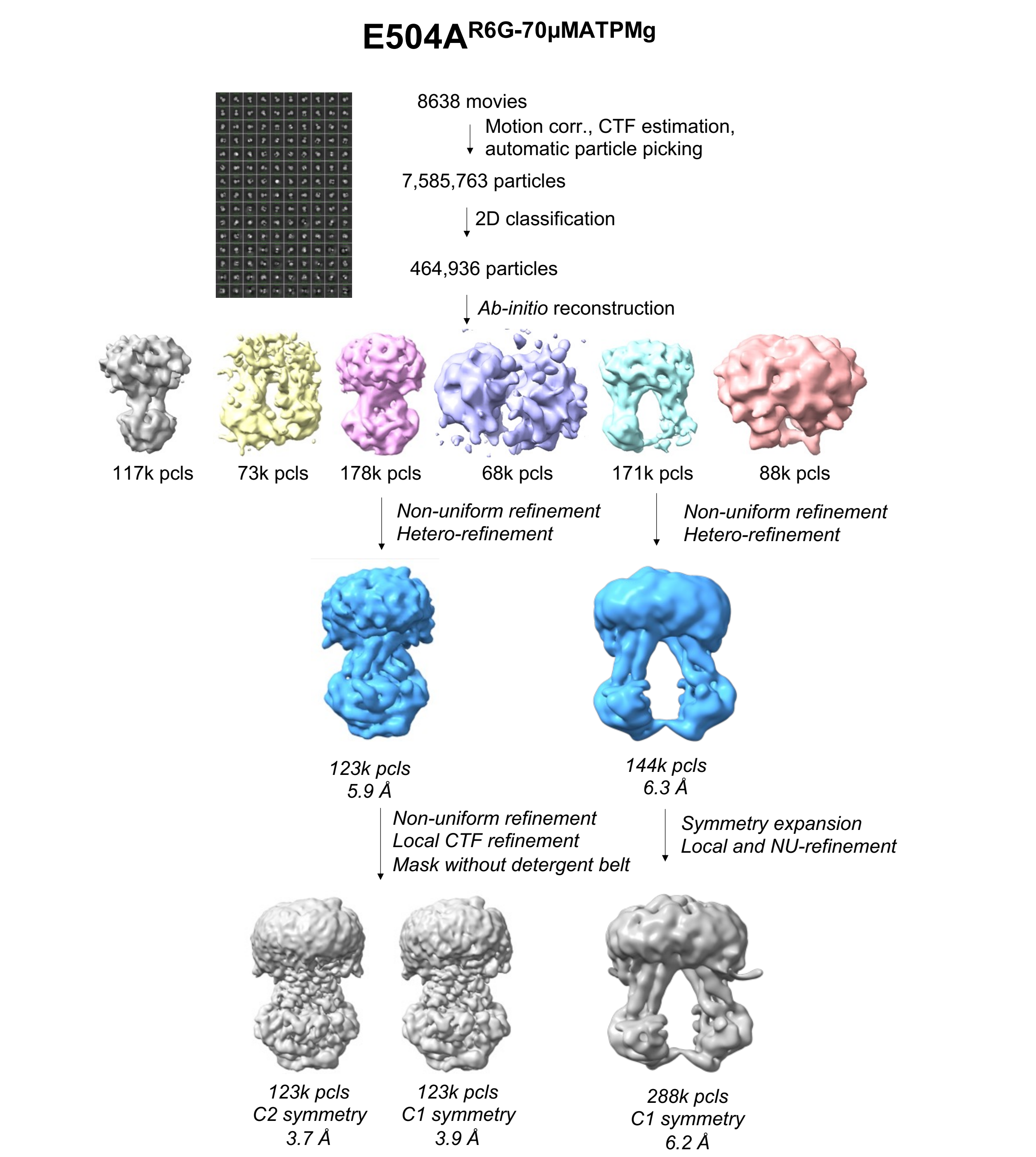

Supp-Figure 6: Data processing scheme for BmrA E504A$^{R6G-70\mu MATPMg}$. Particles (pcls) are listed for each step and class, and resolution at FSC$^{=0.143}$ are listed for the latest stages of refinement. Many routes were explored to reach high resolution reconstructions, only the final one is displayed.

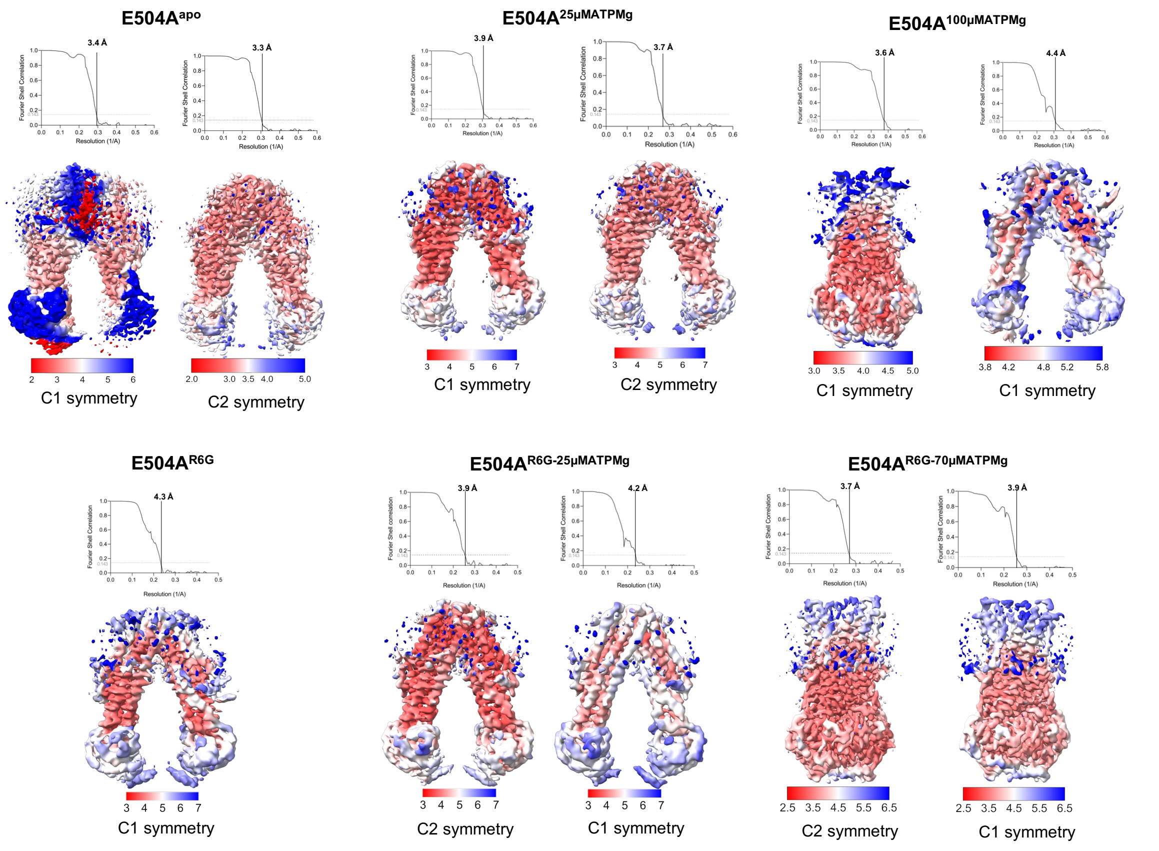

Supp-Figure 7: Local resolutions estimations for each dataset. Without R6G on top, with R6G at the bottom. Resolution scales vary for each dataset to better render the resolution range of each reconstruction.

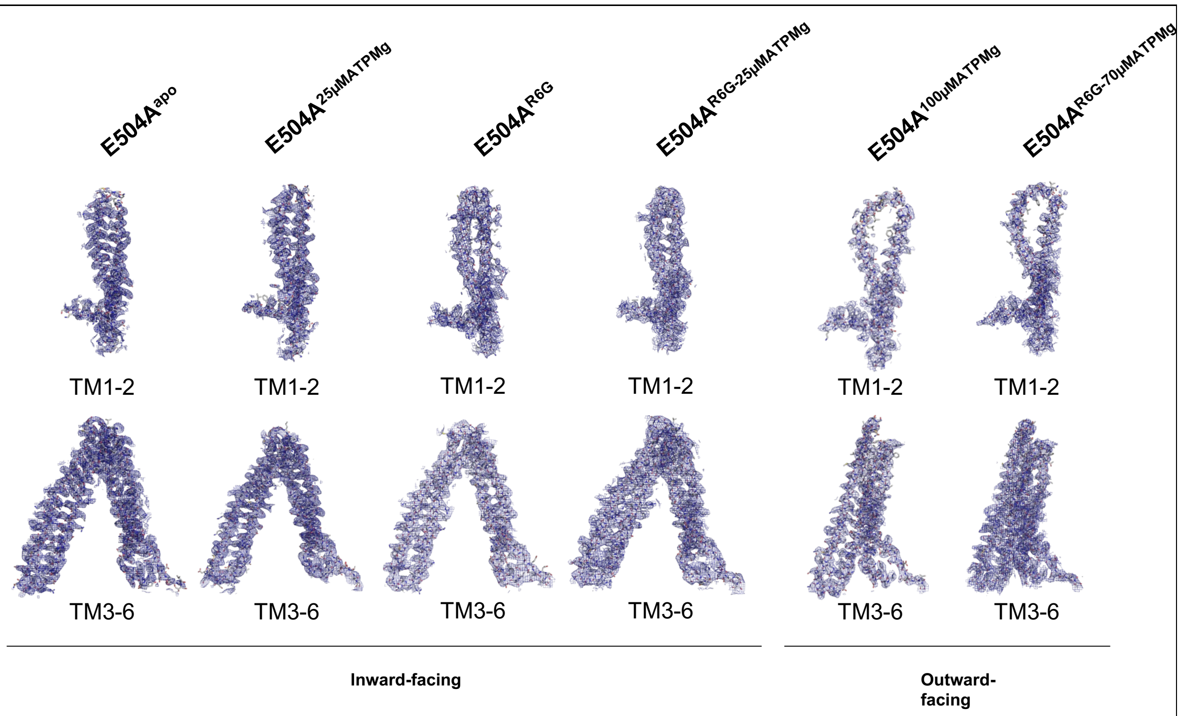

Supp-Figure 8: Coulomb potential maps for the trans-membrane regions of each dataset, exemplifying the backbone and side chains can be placed unambiguously and that the conformational changes induced by substrate binding are indeed reflected by changes in reconstructions.

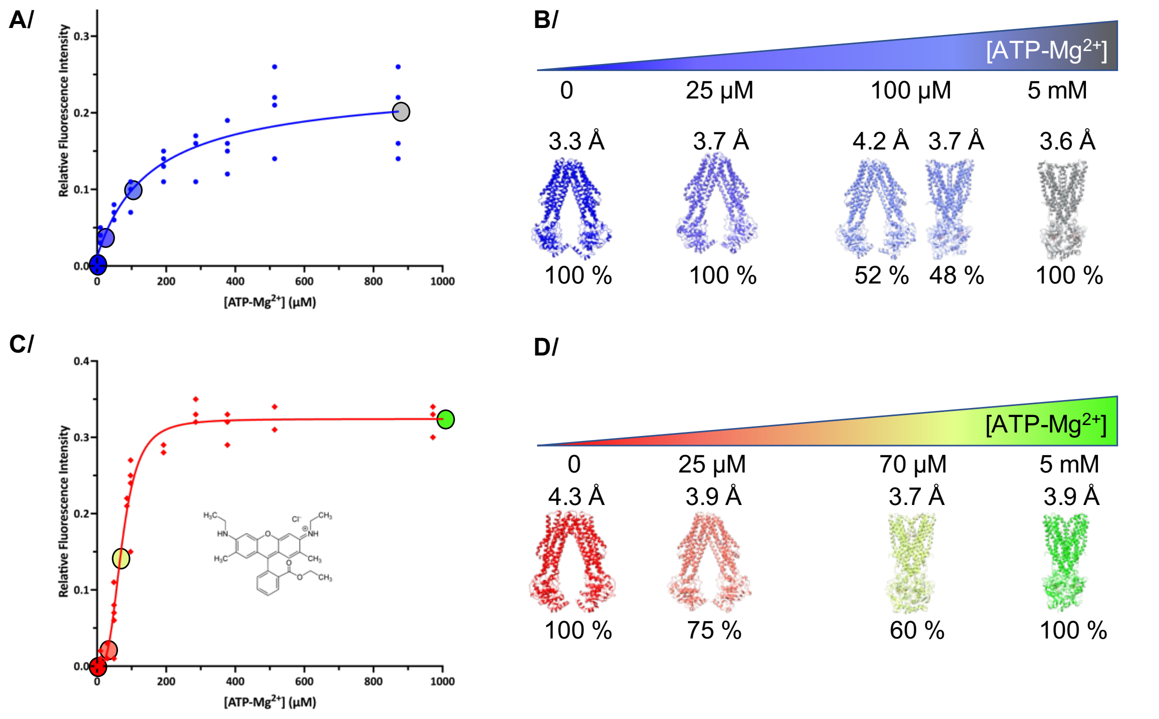

Supp-Figure 9: Models generated for each reconstruction. **A/**, **B/**, **C/** and **D/** as in Figure 1, with the same color code. A/ the data is a quadruplicate, acquired as a duplicate in two different biological replicates. C/ the data is a combination of 3 experimental replicates, with 1 triplicate and 2 duplicates. Models are represented instead of reconstructions in **B/** and **D/**, when resolution was sufficient to generate a model.

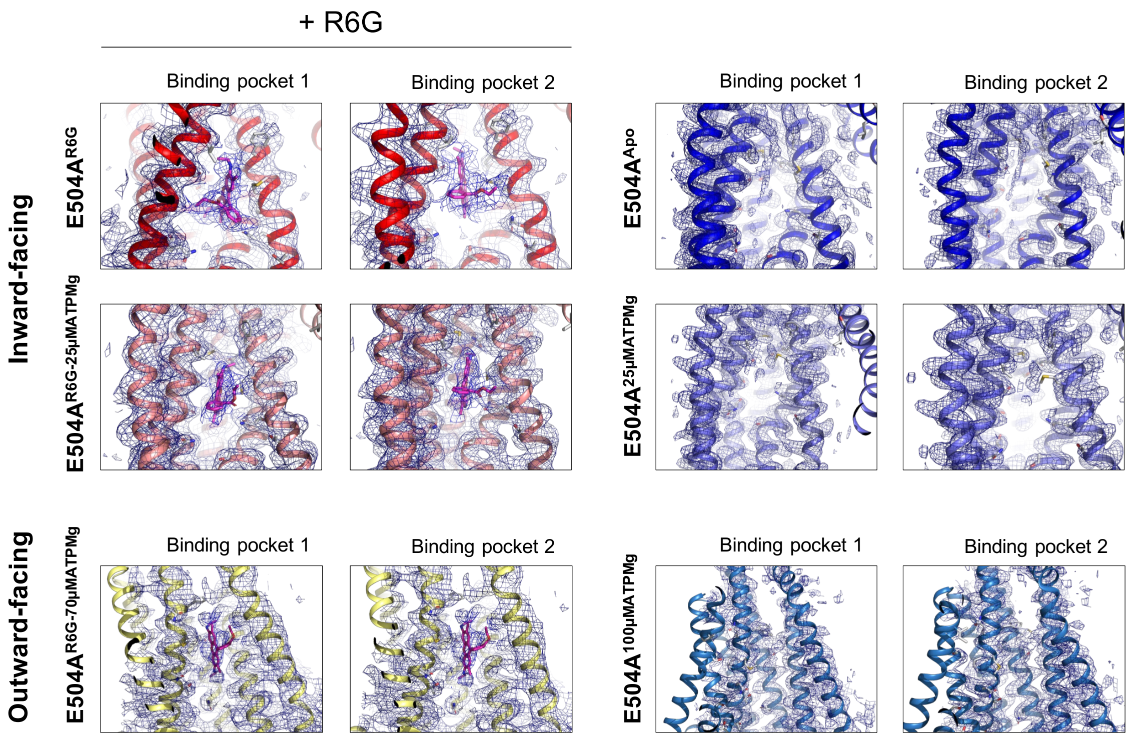

Supp-Figure 10: Coulomb potential maps for R6G in the binding pocket. Left side displays all datasets with R6G present in the solution, right side without R6G. Maps are shown in blue mesh, and the models are displayed in cartoon with the same coloring scheme as in Figure 1. For each dataset, the two halves of BmrA are displayed (binding pockets 1 and 2). R6G is shown as magenta sticks, and its density is indeed present each time R6G is added to the protein.

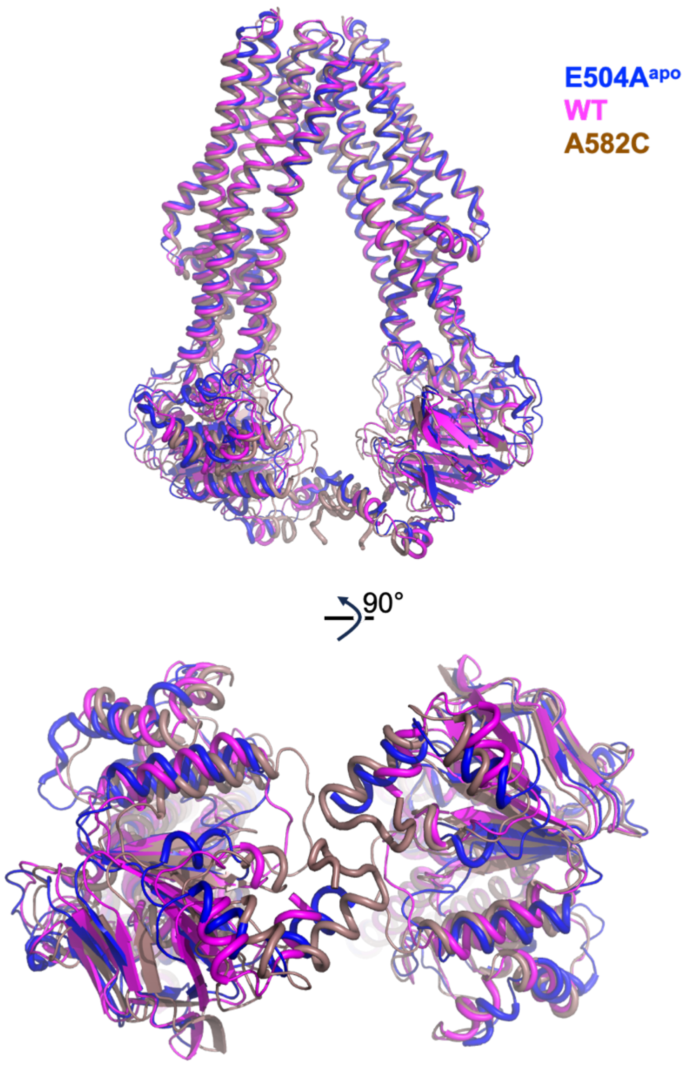

Supp-Figure 11: Overlay of BmrA WT, A582C and E504A in the apo forms. E504A^{apo} is colored in blue according to the color scheme of Figure 1. BmrA WT (PDB code 8qoe) is colored magenta, and the A582C mutant (PDB code 8chb) is colored brown. All proteins are represented in cartoon from the side (top) and from the NBDs (bottom). Overlay performed on residues 1-161 of chain A for each structure with a rmsd of 0.52Å over 902 atoms and 0.45 Å over 883 atoms, respectively.

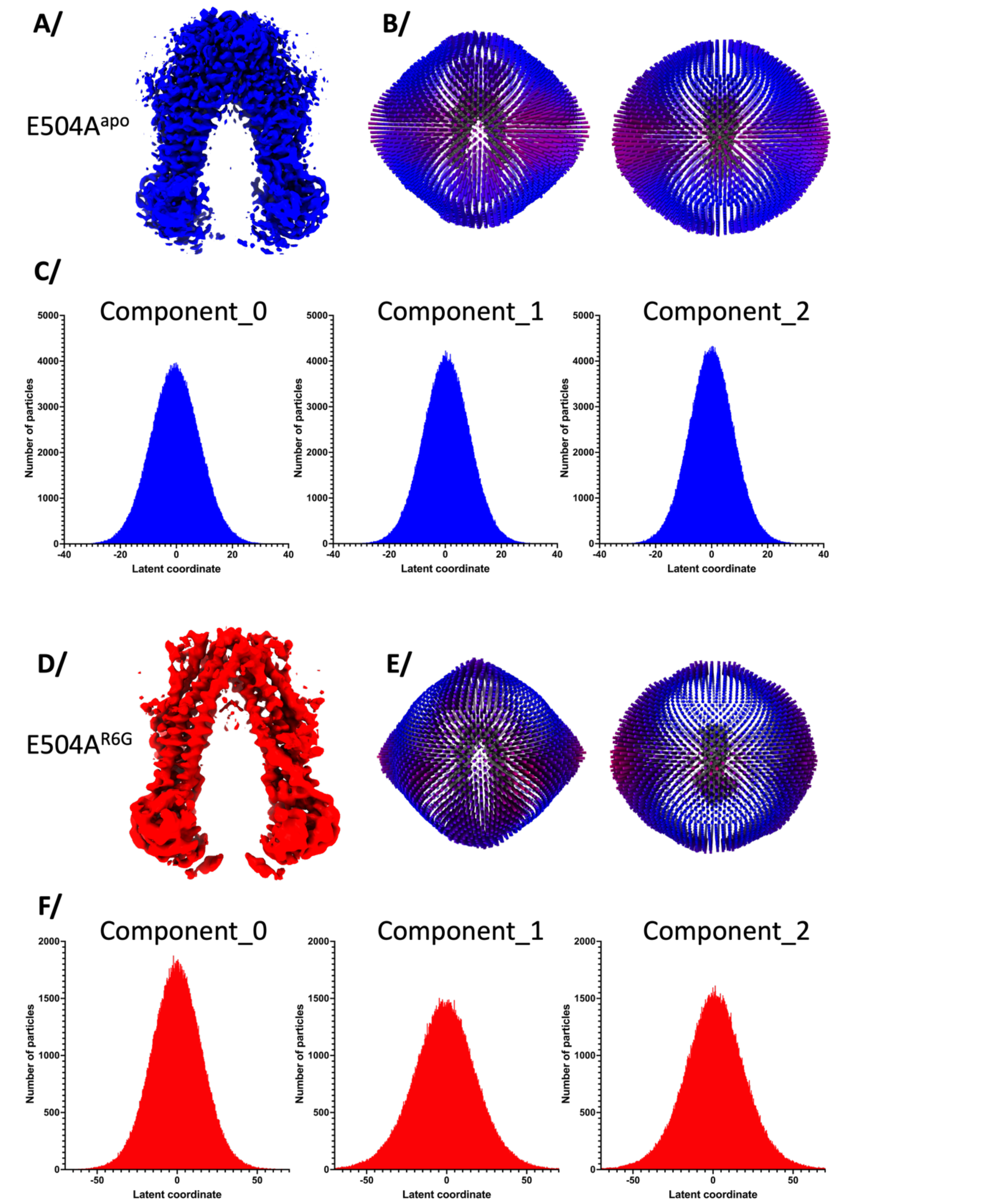

Supp-Figure 12: Particle distributions. **A/** E504^{apo} consensus reconstruction. **B/** 3D representation of particle distribution for the reconstruction; 2 views at 90° are represented. **C/** Particle distribution in latent space, represented per principal component as a function of latent coordinate. **D/ E/ F/** same as above for E504A^{R6G}.

A/

B/

C/

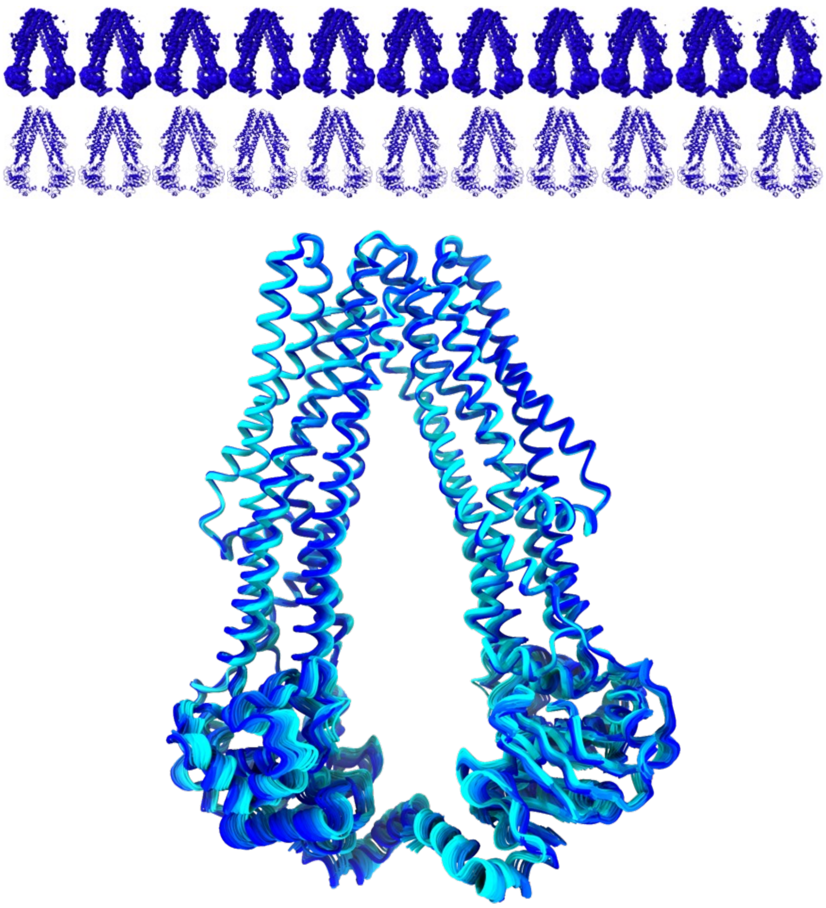

Supp-Figure 13: Explanation of *varref* output from 3DVA analysis. **A/** 11 evenly spaced outputs of the 20 maps from 3DVA analysis in cryosparc for the condition BmrA^{apo}. **B/** In each map, a model was refined by *phenix.varref*. The difference in models can be followed at the lowest part of the structure in C-terminal helices. **C/** All 20 models output from *varref* are shown and colored from blue to cyan to show the movement undergone by the protein.

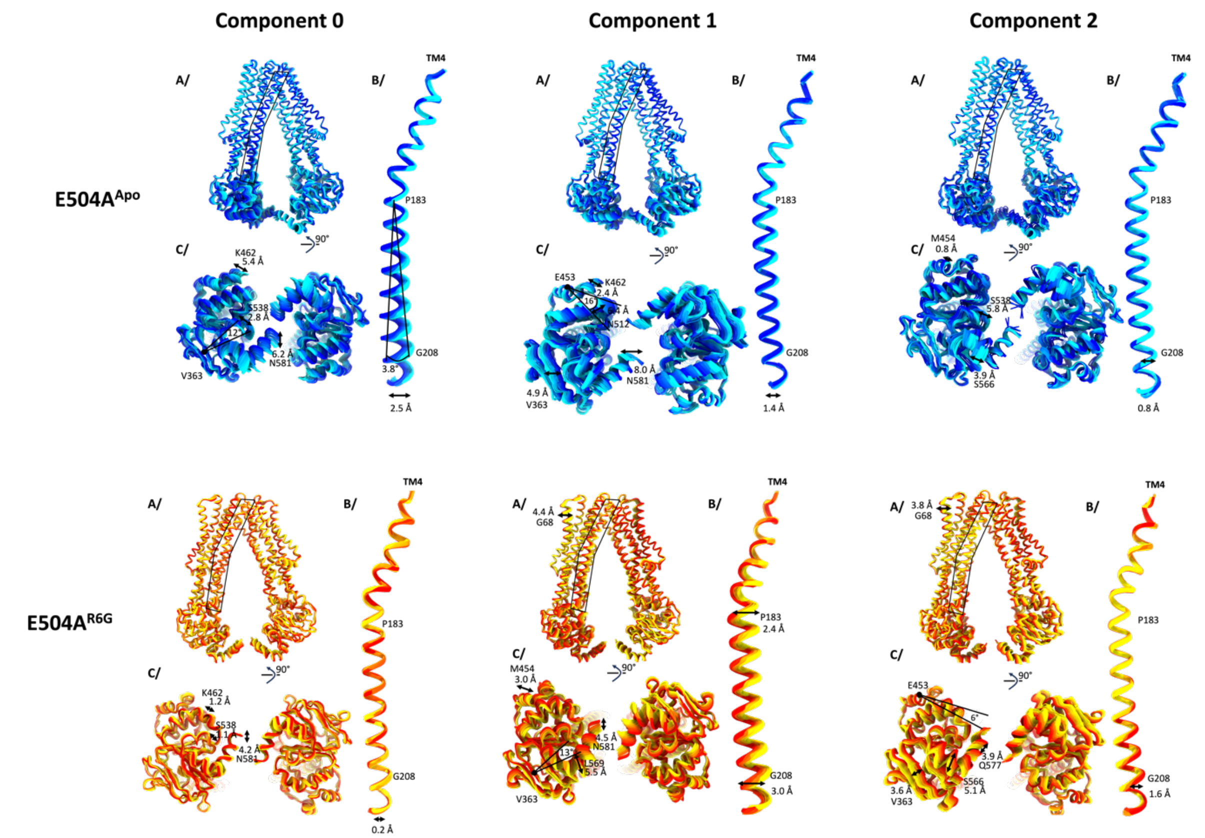

Supp-Figure 14: *varref* analysis for E504A^{apo} and E504A^{R6G}. E504A^{apo} is represented in a gradient of blue to cyan and E504A^{R6G} from red to yellow. For each protein, the movement is decomposed per principal component during the 3DVA analysis. For each panel **A/** is the lateral view of the full transporter, **B/** is the zoom on TM4, and **C/** is the view of the NBD from under. On each sub-panel, the rotation or translation is displayed and measured.

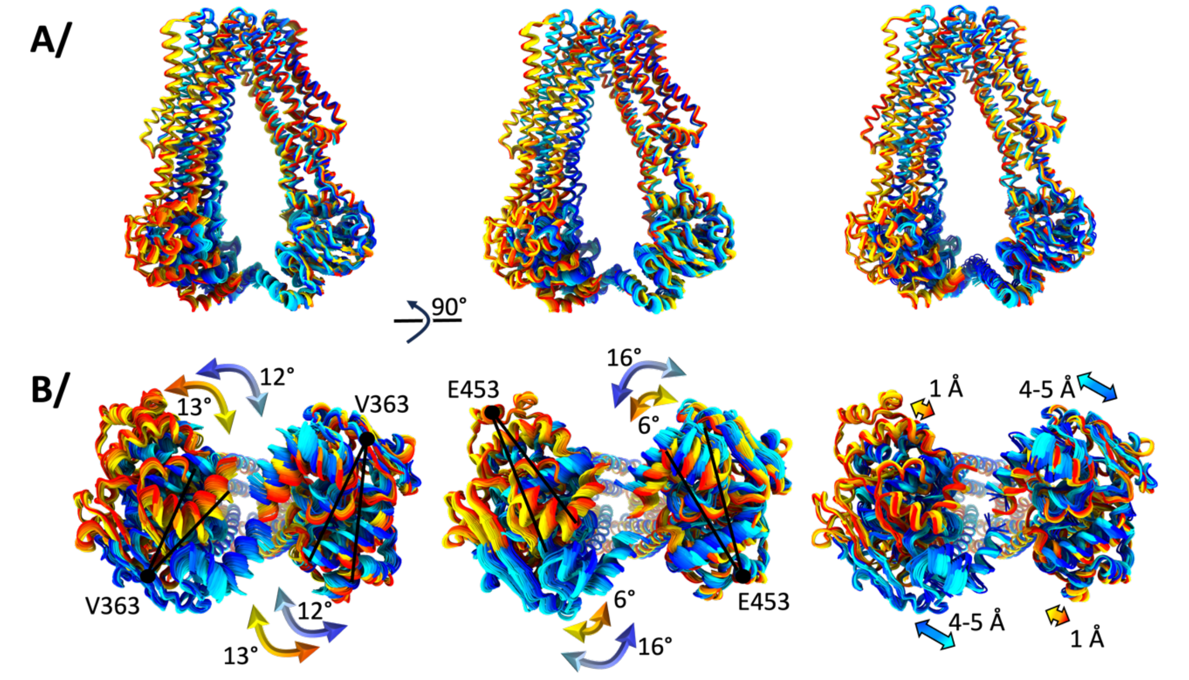

Supp-Figure 15: *varref* analysis for E504A^{apo} and E504A^{R6G}. Same analysis as Supp-Figure 14 this time overlaid. **A/** lateral view and **B/** view from underneath. E504A^{apo} colored from blue to cyan and E504A^{R6G} colored from red to yellow.

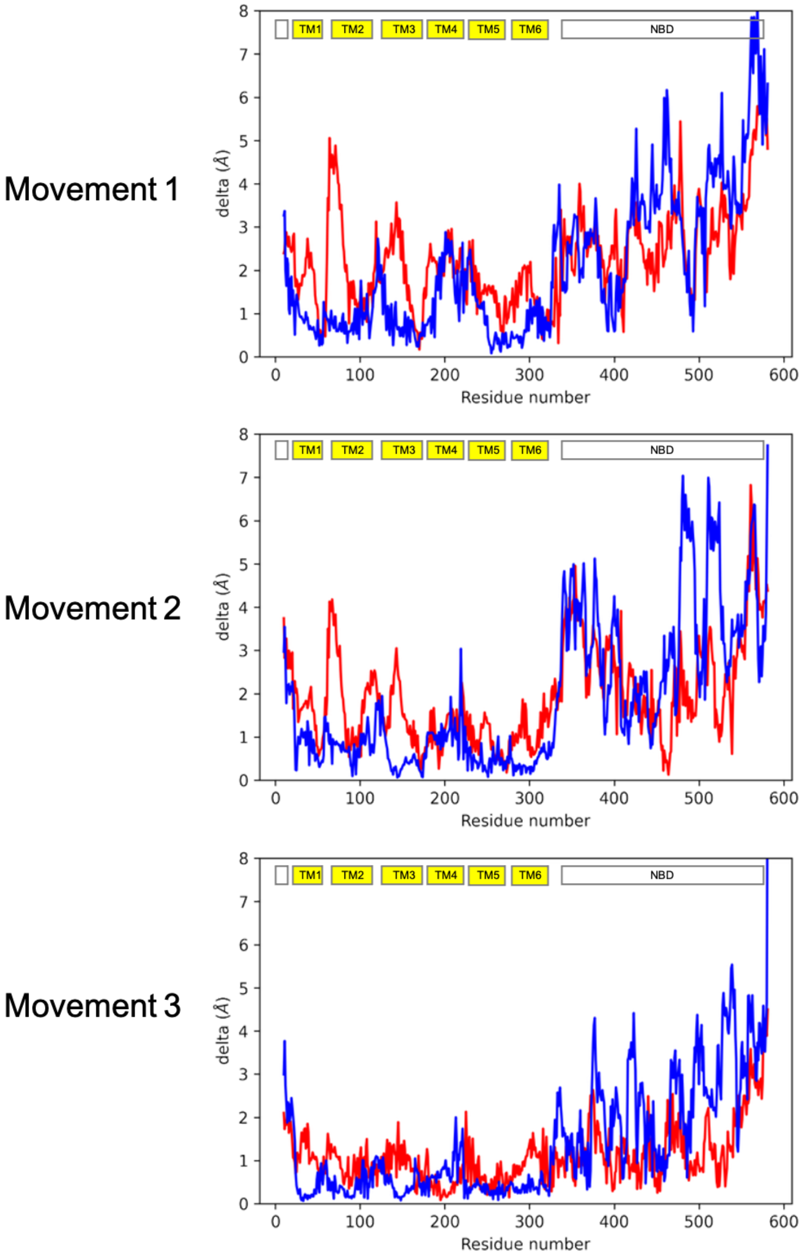

Supp-Figure 16: Structural variations within varref bundles. For each movement described in Figure 3A, rmsf was calculated for each structure within the bundle and plotted as a function of residue number in the chain. An average was performed between chain A and B of the homodimer. BmrA^{apo} is colored blue and BmrA^{R6G} is colored red. The position of each structural element is depicted on top of each graph for reference.

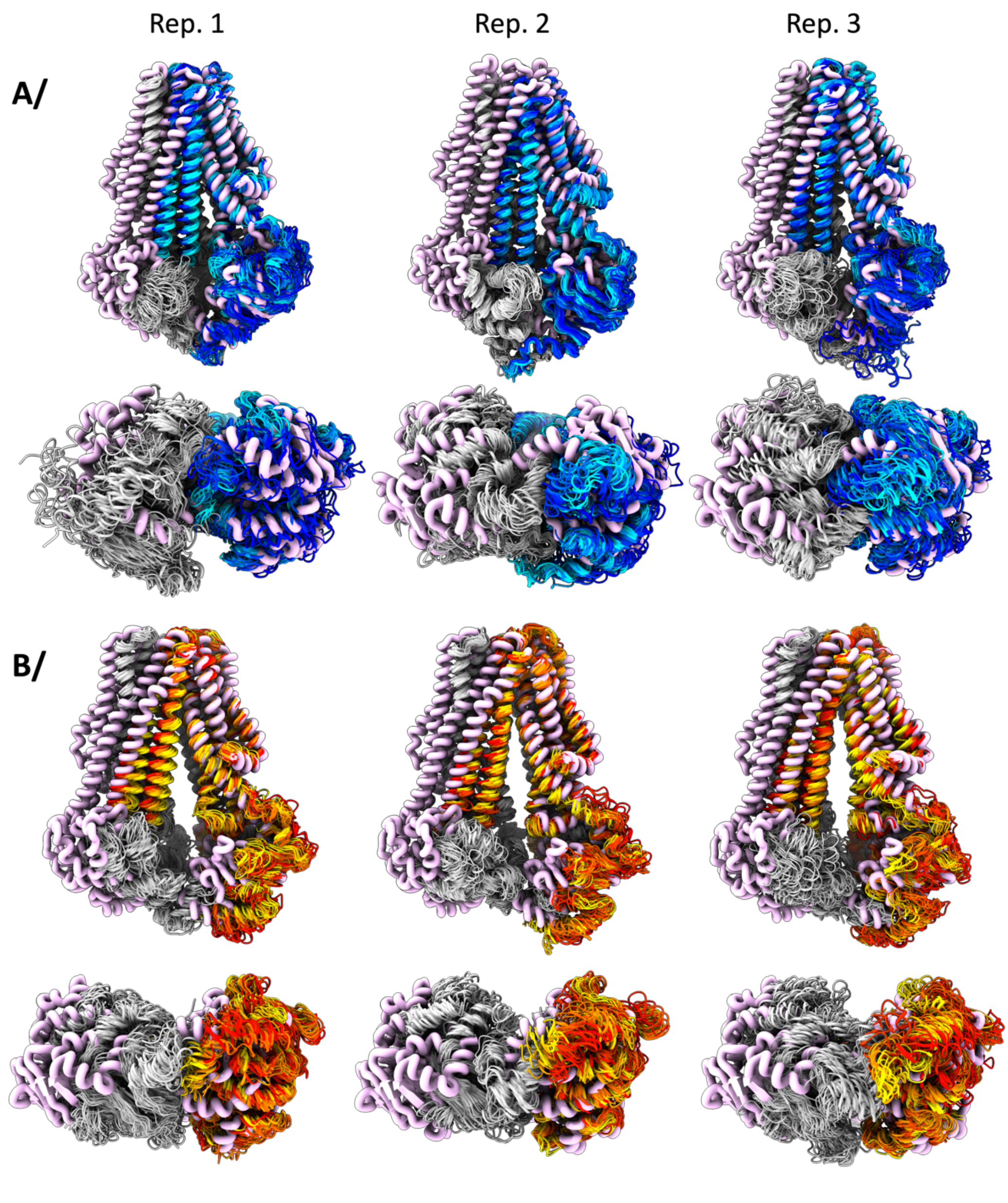

Supp-Figure 17: Visualization of protein movement during each MD simulation. **A/** E504A^{apo} was simulated in 3 independent replicas. The initial structure is shown as thick pink cartoon and a snapshot of the simulation is shown each 20 ns, colored from blue to cyan as the simulation progresses. **B/** Same as A/ but for BmrA^{R6G}, colored from red to yellow.

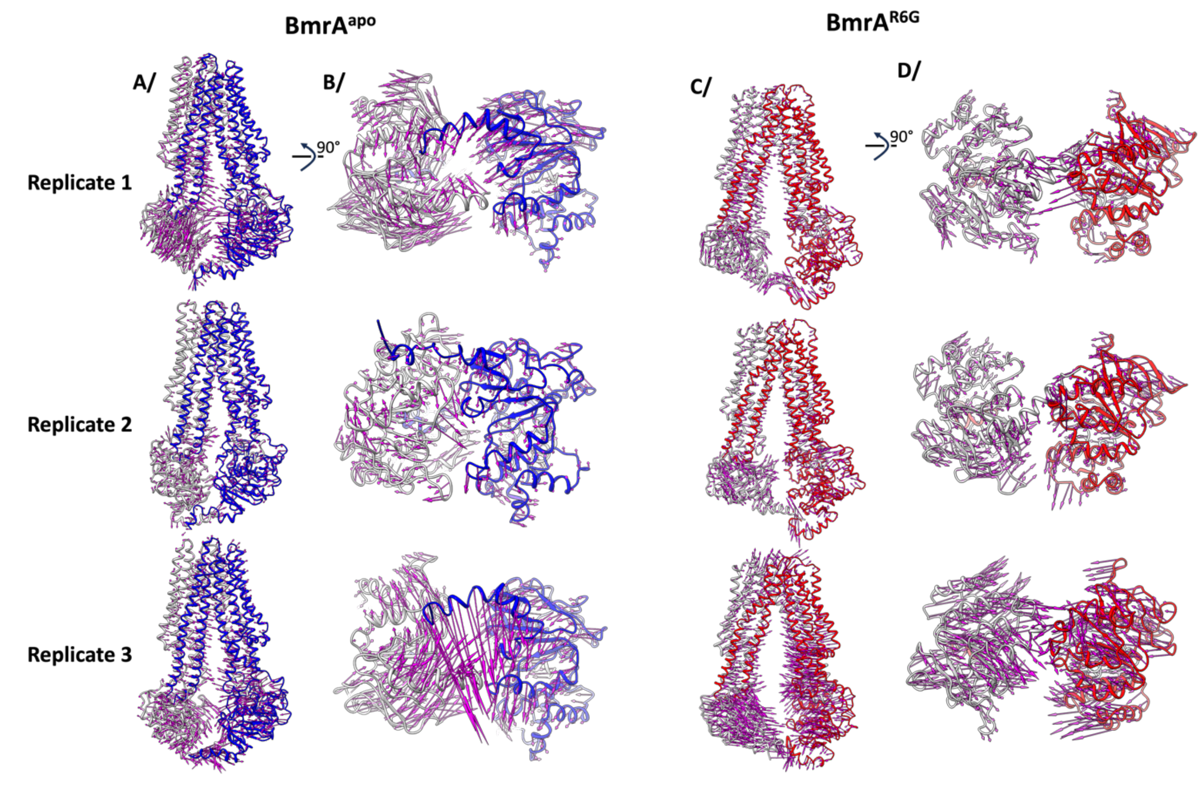

Supp-Figure 18: Visualization of protein movement during MD simulations. Same simulations as supp-Figure 16, with the arrow representing the distance between the initial and final frame of the simulation for E504A^{apo}. One monomer is colored in blue, the other in light grey **A/** Seen from the side, and **B/** seen from under. **C/** and **D/** are the same as A/ and B/ for E504A^{R6G}.

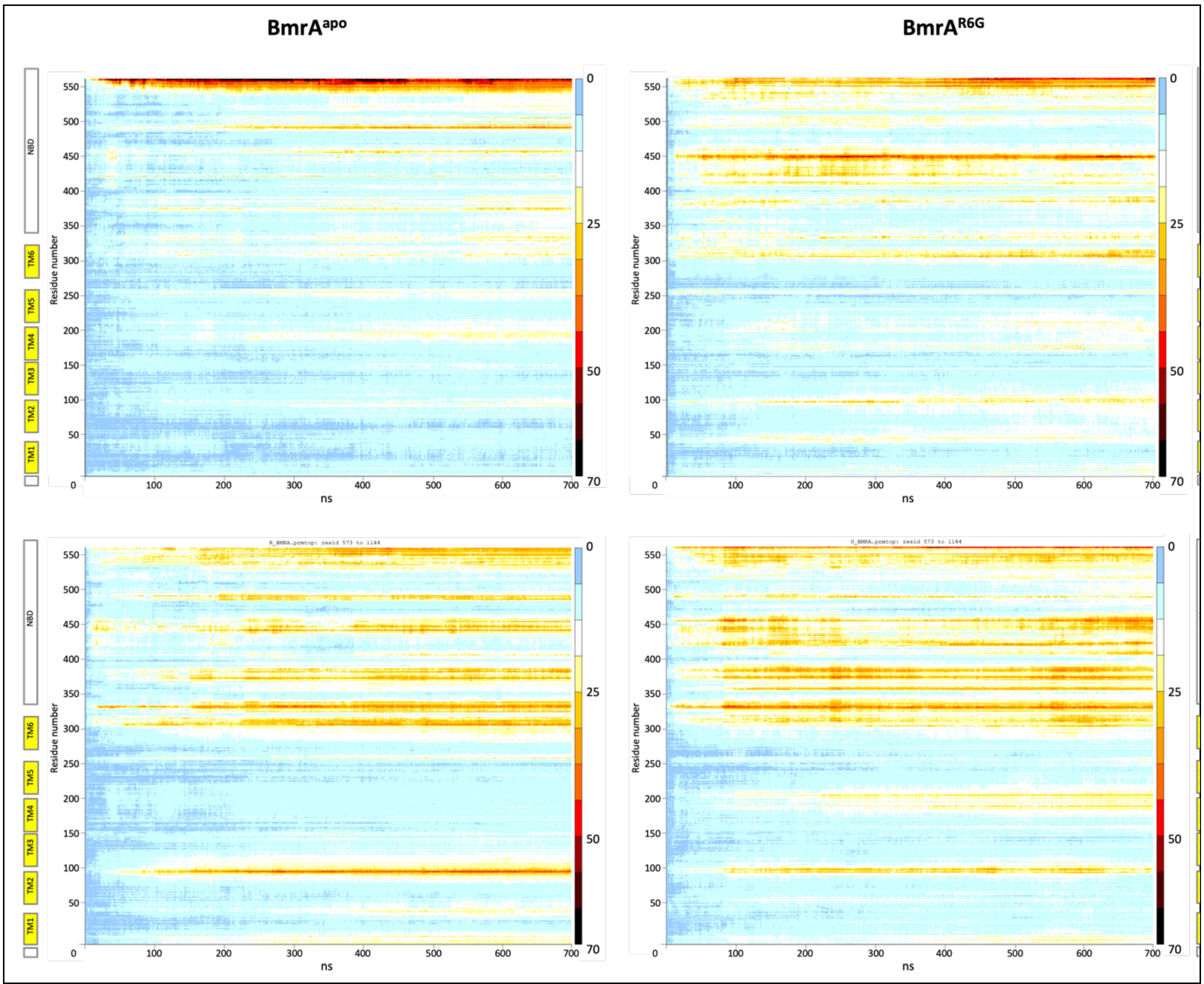

Supp-Figure 19: Residue displacement during MD simulations. For each protein E504A^{apo} or E504A^{R6G}, the sum of displacement by residue across the three dynamics is displayed as a function of time, the initial frame being the reference structure. A frame is displayed every ns, for a total of 700ns for each production simulation. The color scale on the right represents the amount of displacement in Å, from 0 (blue) to 70 Å (black). The top and bottom panels represent the two halves of the transporter.

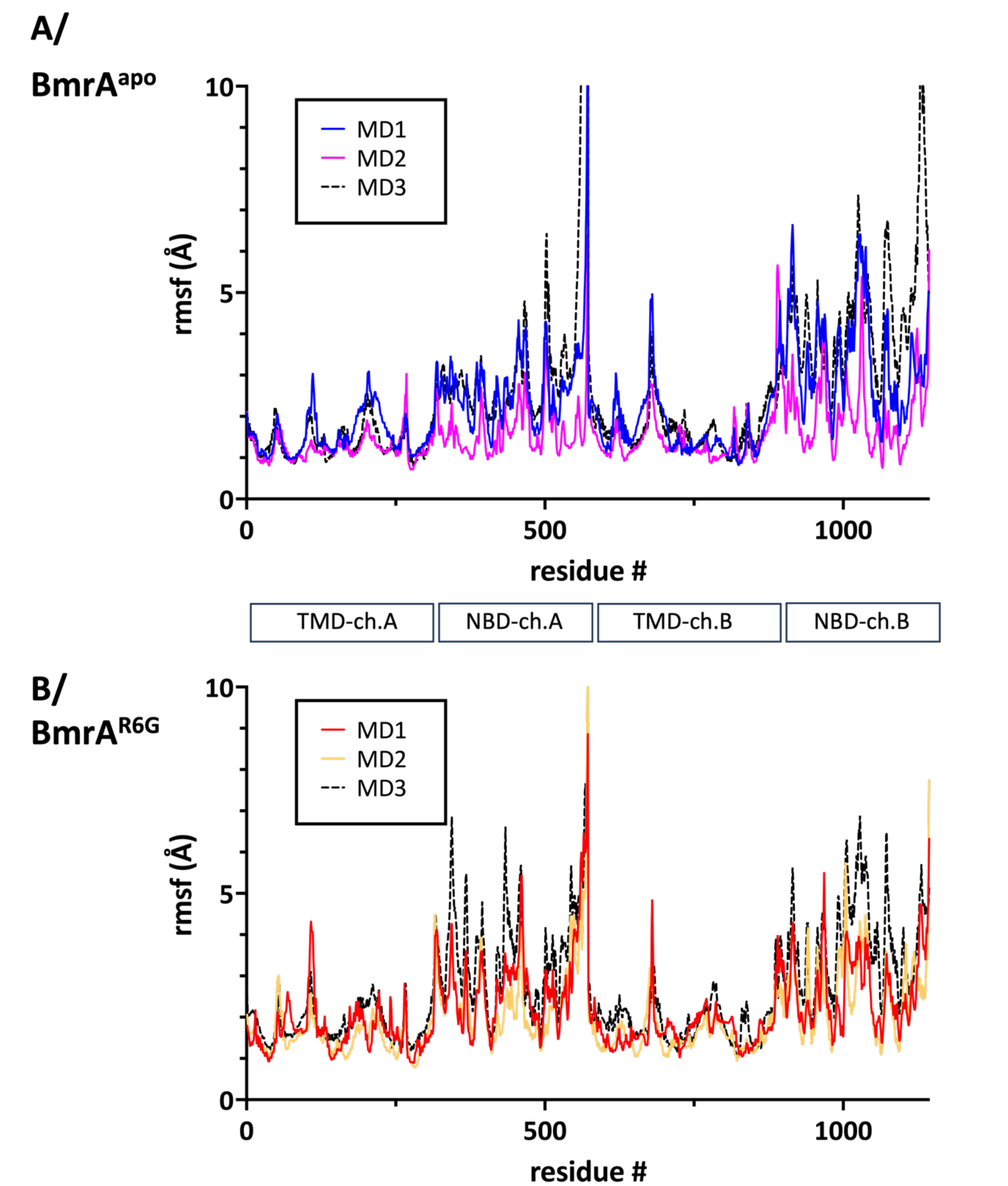

Supp-Figure 20: Residue fluctuations during MD simulations. For each protein **A/** E504A^{apo} or **B/** E504A^{R6G} and each replicate, the per-residue rmsf was calculated on the averaged structure. The BmrA dimer is displayed in each replicate with the TMD and NBD of chains A and B labeled.

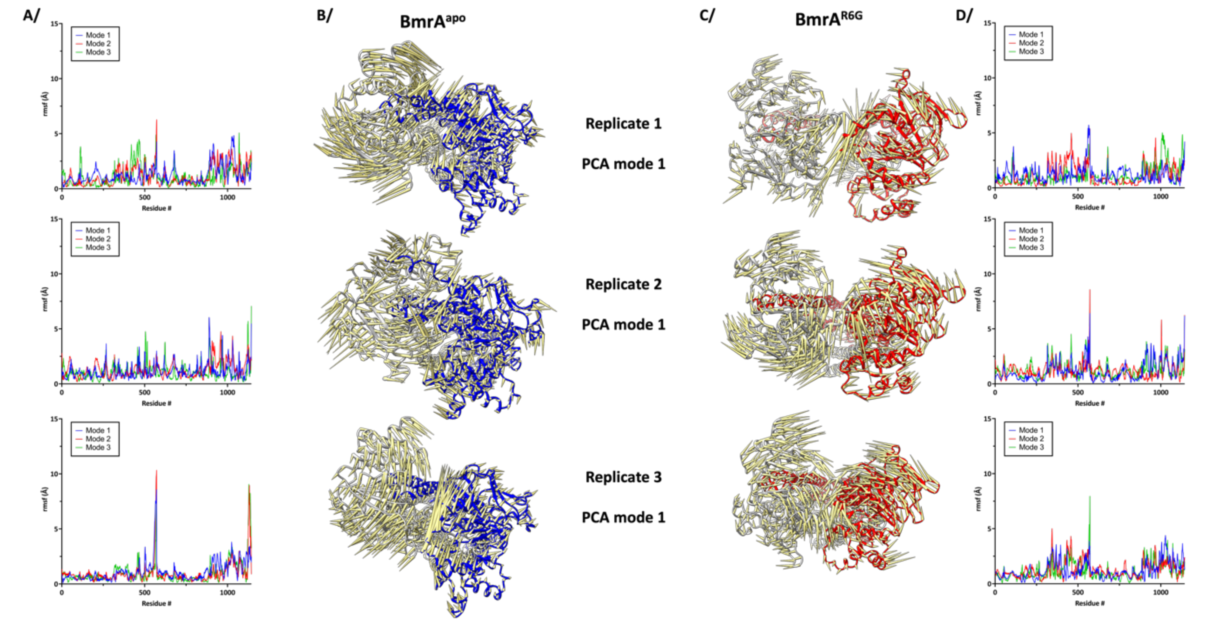

Supp-Figure 21: Principal Component Analysis of MD trajectories. For each protein E504A^{apo} or E504A^{R6G} and each replicate, MD trajectories were subjected to PCA analysis. **A/** rmsf of the PCA analysis over the protein range, for the first 3 modes of the analysis. (TMD from residues 1 to 300 and 590 to 890. NBD from residues 300 to 580 and from 890 to 1140). **B/** Visualization of the direction of the movement for the first mode of the PCA analysis. The initial structure is displayed in cartoon, blue for one chain, silver for the other one. Yellow cones represent the direction of the movement of this mode. **C/** same analysis as B/ for E504A in presence of R6G. **D/** Same analysis as A/ for E504A in presence of R6G.

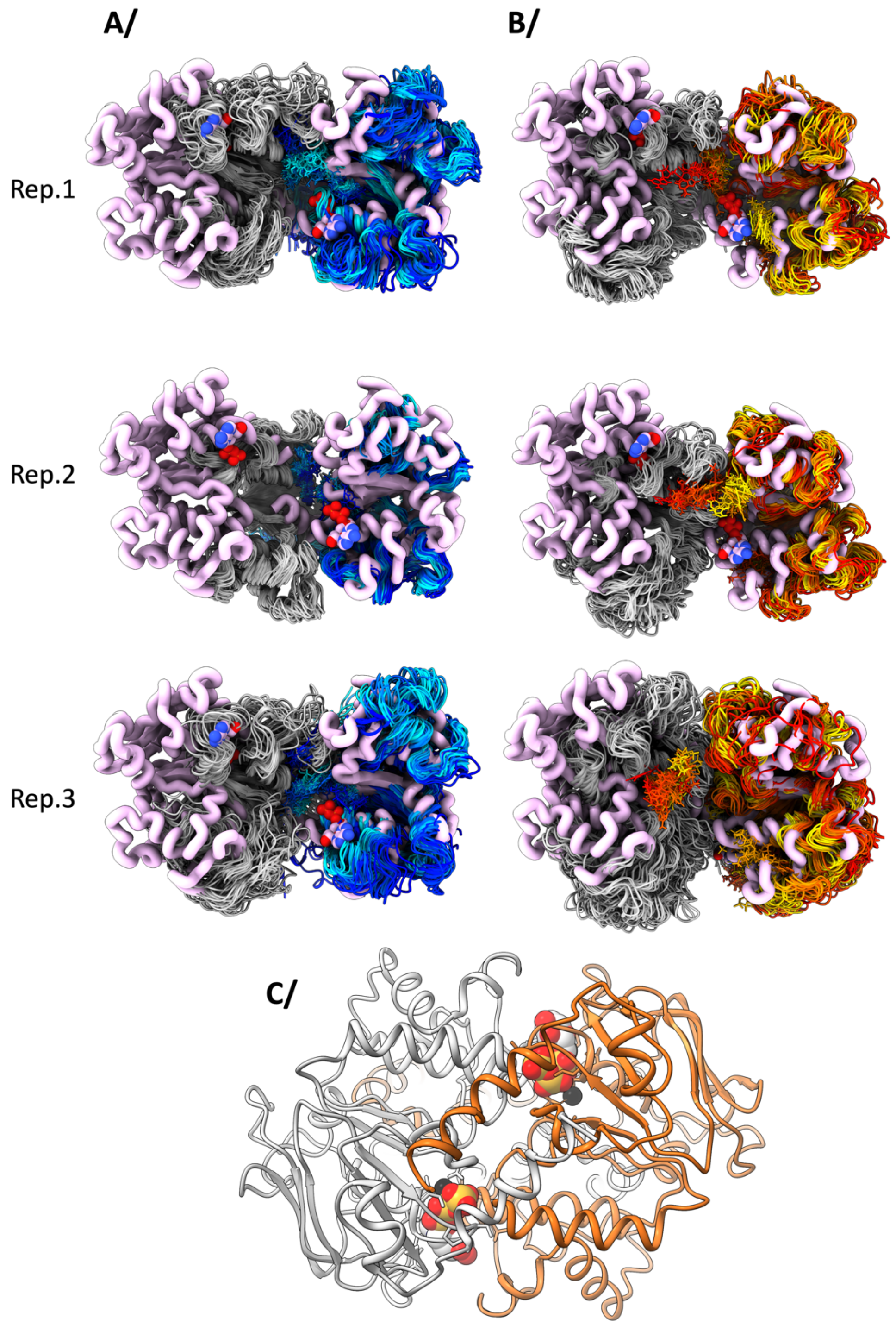

Supp-Figure 22: NBD and ATP flexibility during MD simulations. **A/** BmrA^{apo}. **B/** BmrA^{R6G}. For each replicate, the initial structure is shown in thick pink cartoon and initial ATP-Mg^{2+} position in thick sticks colored by atom type. The simulation of 700ns has been divided in 35 snapshots separated at equal time during the simulation, and represented in cartoon for the protein and sticks for ATP-Mg^{2+}. The colors for the simulation range from blue to cyan or red to yellow to match the observations by 3DVA. **C/** OF conformation of BmrA for reference (PDB 7bg4), one monomer in orange, the other silver, ATP in spheres colored by atom type, and Mg in black.

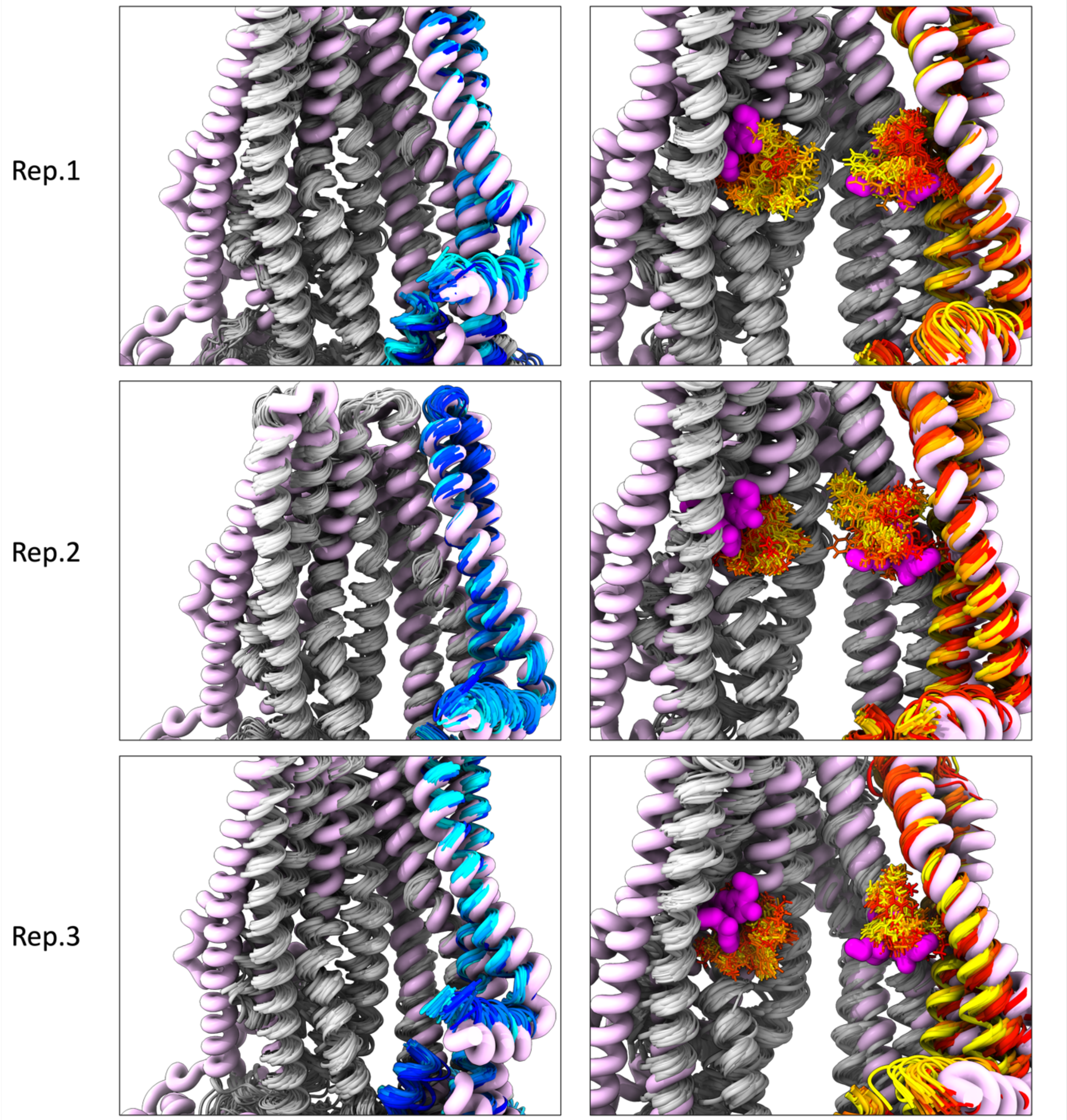

Supp-Figure 23: Mobility of R6G in its binding pocket. For each replicate of $BmrA^{R6G}$, the initial structure is shown in thick pink cartoon and initial R6G position in magenta thick sticks. The simulation of 700ns has been divided in 35 snapshots separated at equal time during the simulation, and represented in cartoon for the protein and sticks for R6G. The colors for the simulation range from red to yellow to match the observations by 3DVA. The 3 replicates were made. Left panel, no R6G was present.

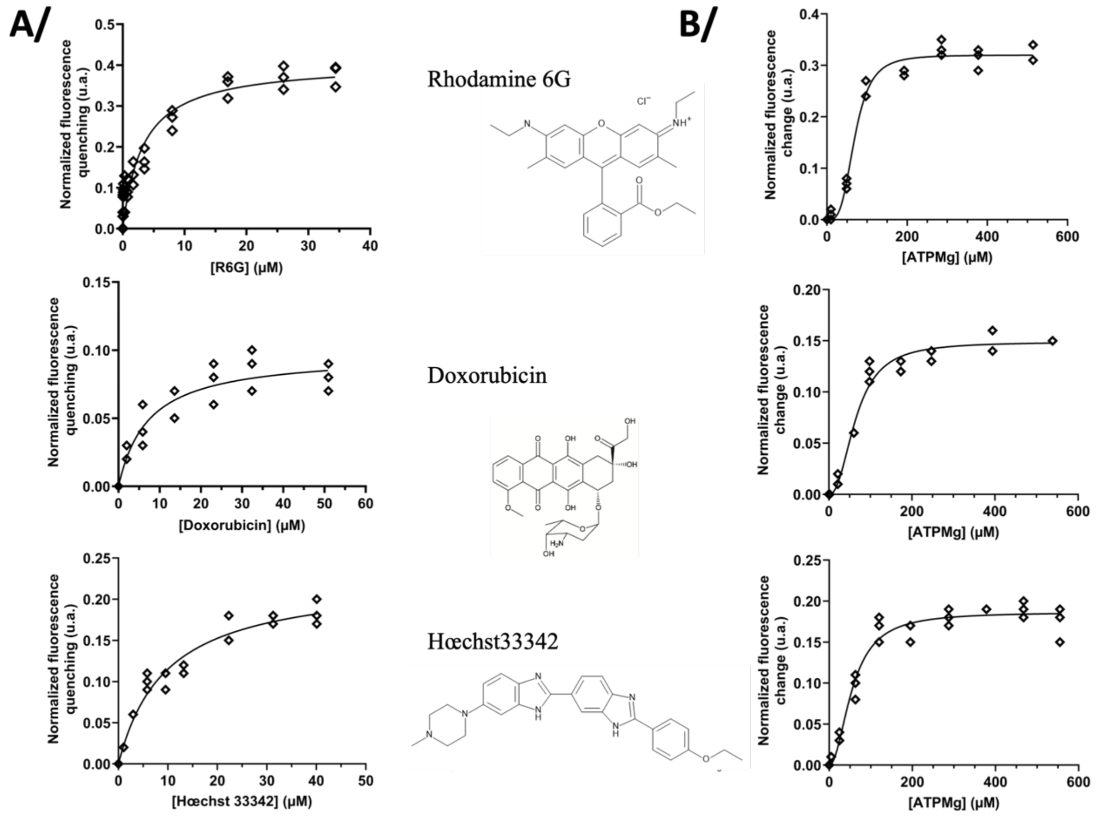

	Drug		ATP		
**Ligands**	**Kd Drug (µM)**	**F_{max} (u.a.)**	**$K_{0.5}$ ATP (µM)**	**h**	**F_{max} (u.a.)**
**Rhodamine 6G (R6G)**	3.3 ± 0.9	0.41 ± 0.02	70.0 ± 2.6	3.4 ± 0.3	0.32 ± 0.01
**Doxorubicin (Doxo)**	7.3 ± 2.2	0.09 ± 0.01	65.5 ± 3.6	2.3 ± 0.3	0.15 ± 0.01
**Hœchst 33342 (H33342)**	9.6 ± 1.3	0.23 ± 0.01	56.0 ± 4.0	2.0 ± 0.3	0.19 ± 0.01

Supp-Figure 24: Drug and ATP binding on BmrA-E504A. Binding of R6G, Doxorubicin or Hœchst33342 were measured using intrinsic fluorescence displacement Normalized fluorescence quenching is presented. B/ ATP-Mg^{2+} binding on BmrA-E504A in presence of drug, measured by intrinsic fluorescence (Relative fluorescence increase is observed). The table recapitulates the K_d (drug), $K_{0.5}$ (ATP), Hill number (h) and the maximum fluorescence quenching (F_{max}). Experiments were carried in an experimental triplicate.

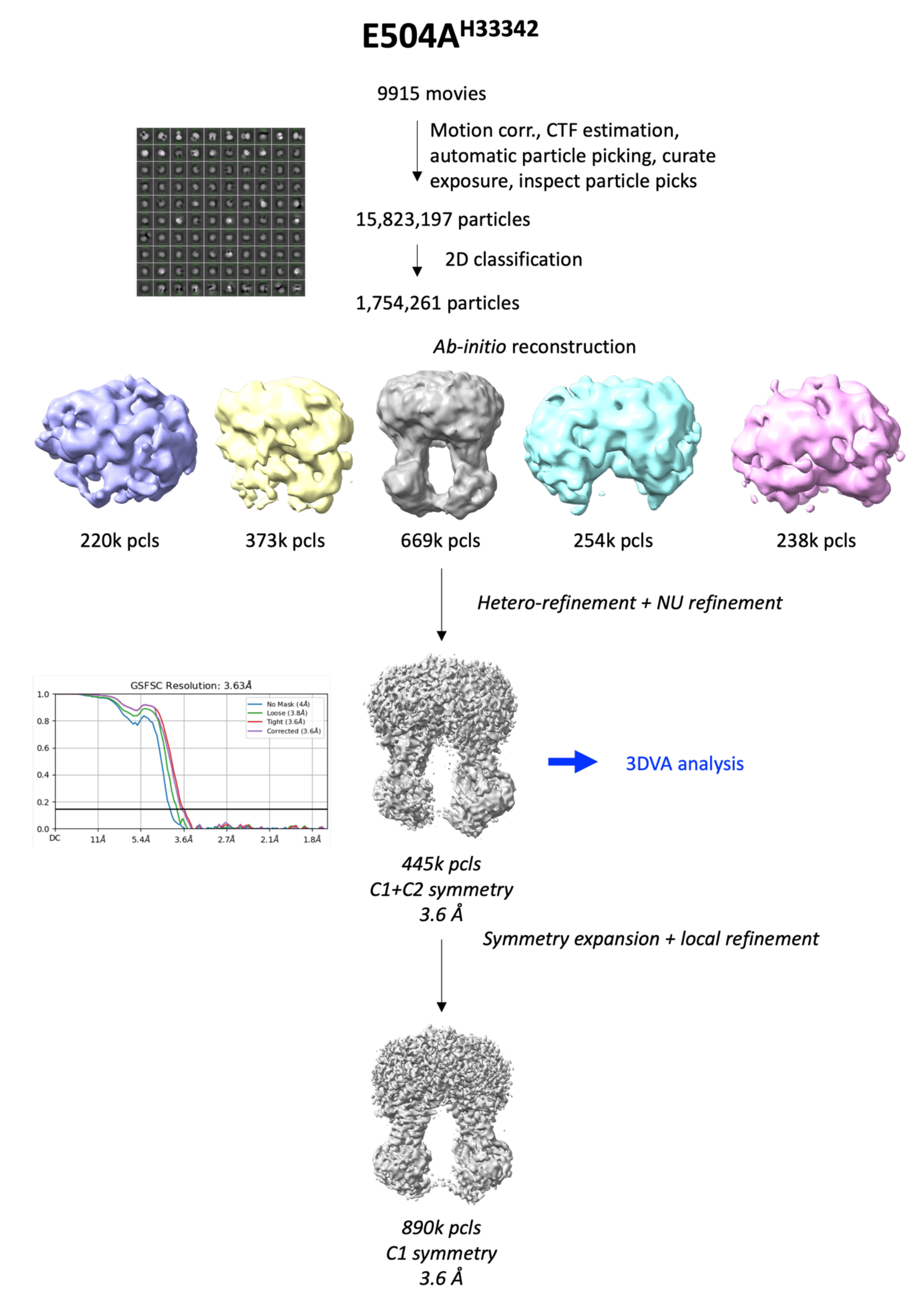

Supp-Figure 25: Data processing scheme for BmrA E504A^{H33342}. Particles (pcls) are listed for each step and class, and resolution at FSC$^{=0.143}$ are listed for the latest stages of refinement. Many routes were explored to reach high resolution reconstructions, only the final one is displayed. Final FSC curve shown on the side.

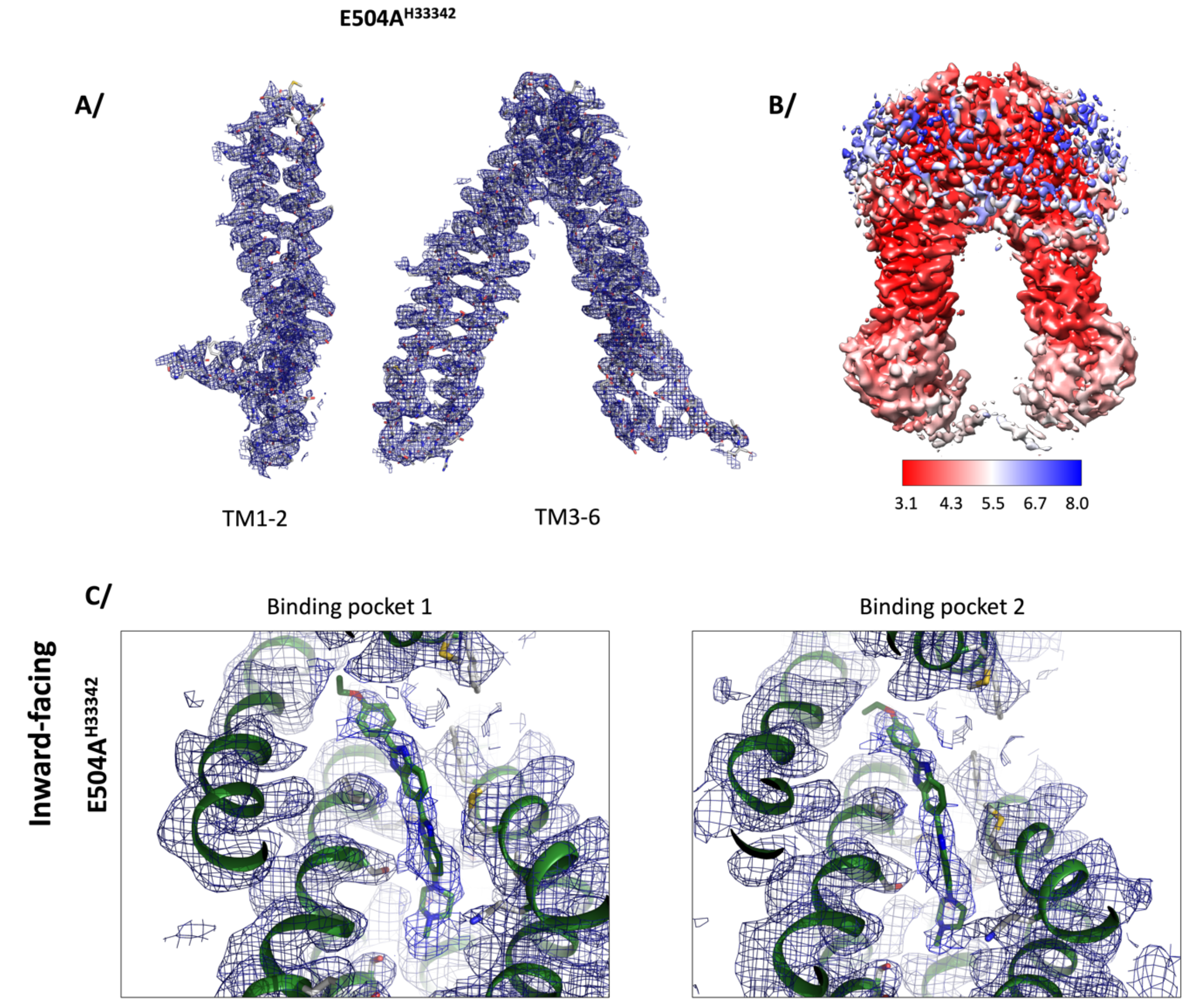

Supp-Figure 26: Coulomb potential maps for E504AH^{33342}. **A/** Maps for the trans-membrane regions. **B/** Local resolution of the coulomb potential map colored from red to blue with the resolution in Å listed below the horizontal bar. **C/** Zoom on the Hœchst33342 binding site. The protein is represented as green cartoon and the Hœschst33342 in sticks colored by atom type with carbons is green. The maps used to better visualize density for the ligand is the one originating from symmetry expansion.

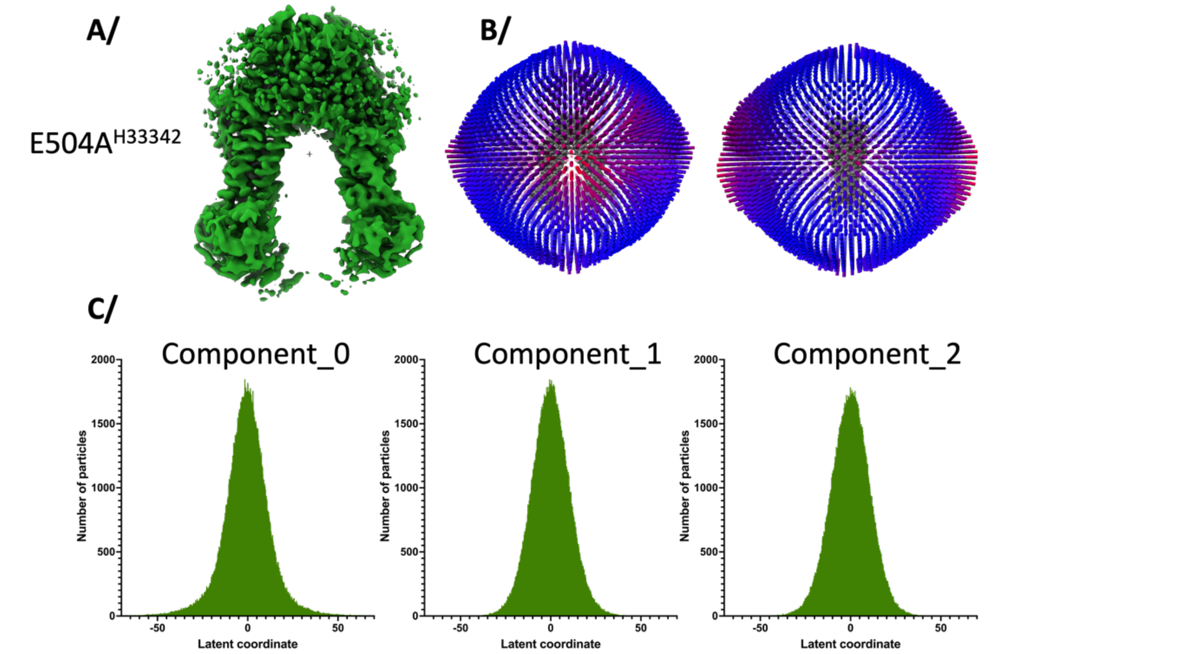

Supp-Figure 27: Particle distributions. **A/** E504^{H33342} consensus reconstruction. **B/** 3D representation of particle distribution for the reconstruction; 2 views at 90° are represented. **C/** Particle distribution in latent space, represented per principal component as a function of latent coordinate.

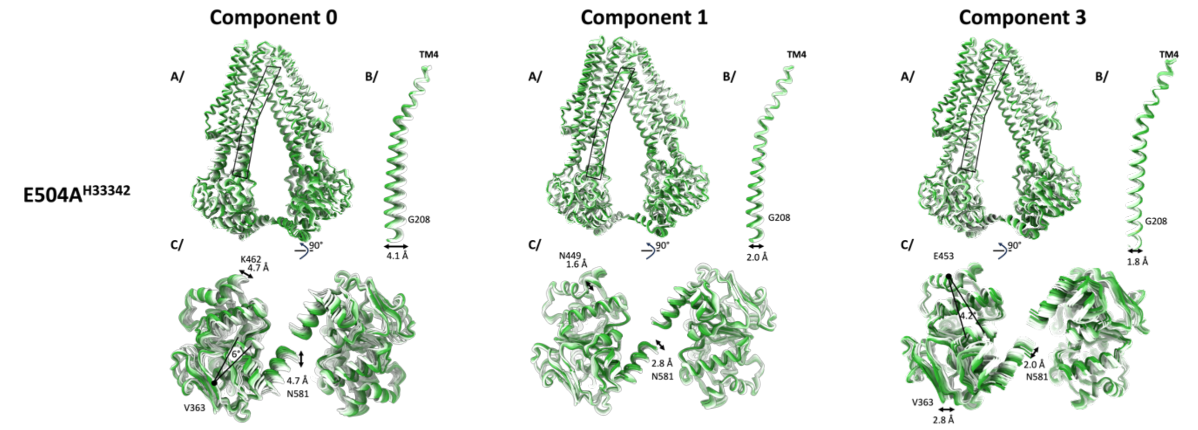

Supp-Figure 28: *varref* analysis for E504A^{H33342}. E504A^{H33342} is represented in a gradient of green to white. The movement is decomposed per principal component during the 3DVA analysis. For each panel **A/** is the lateral view of the full transporter, **B/** is the zoom on TM4, and **C/** is the view of the NBD from under. On each sub-panel, the rotation or translation is displayed and measured.

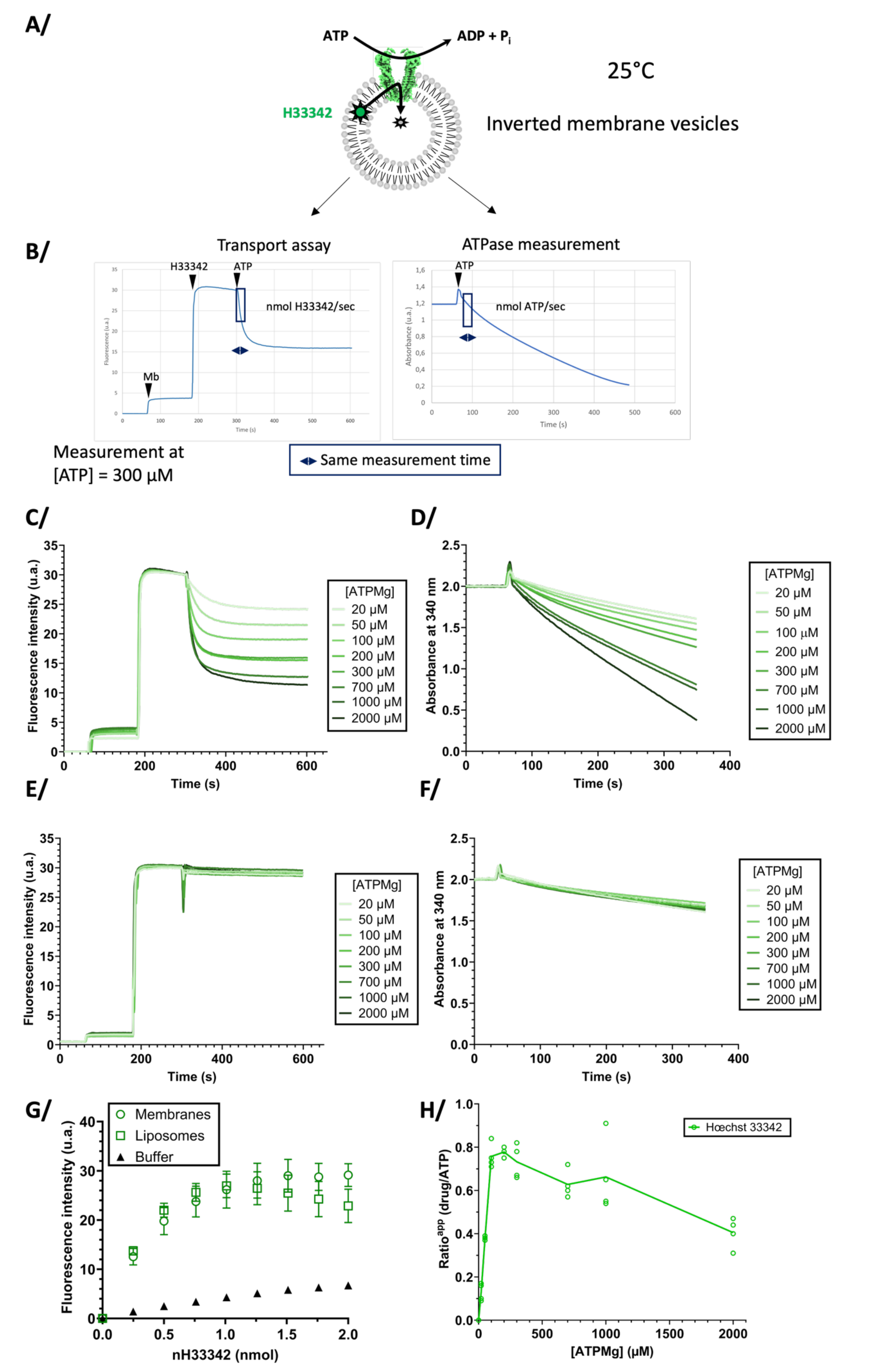

A/
ATP
ADP + Pi
25°C
H33342
Inverted membrane vesicles
B/
Transport assay
ATPase measurement
H33342
ATP
Mb
nmol H33342/sec
nmol ATP/sec
Measurement at
[ATP] = 300 µM
Same measurement time
C/
D/
Fluorescence intensity (u.a.)
Absorbance at 340 nm
Time (s)
[ATPMg]
20 µM
50 µM
100 µM
200 µM
300 µM
700 µM
1000 µM
2000 µM
E/
F/
G/
H/
Membranes
Liposomes
Buffer
nH33342 (nmol)
Ratio^{app} (drug/ATP)
[ATPMg] (µM)
Hœchst 33342

Supp-Figure 29: transport of Hœchst 33342 and ATPase activity for BmrA WT. **A/** Schematic representation of the experiment. **B/** Representative traces of substrate transport (left) and ATPase activity (right) for BmrA WT performed on the same membrane vesicles, the same day. All experiments have been carried out in quadruplicates, from 2 different membrane batches. The time range of measurement for transport is shown as a black rectangle on each graph. **C/** One monoplicate of substrate transport for the whole range of ATP tested. **D/** corresponding ATPase activities on the same membranes. C/ and D/ have been measured in quadruplicate available in source data, being 4 biological replicates. **E/ F/** same as C/ and D/ but for the inactive mutant E504A. Contaminant activities measured with the mutant E504A were deduced to BmrA WT activities for both transport and ATPase activities. **G/** Standard curve of Hœchst33342 fluorescence increase as a function of Hœchst33342 being added to a fixed amount of lipid, done on membranes (green circles), liposomes (green squares; liposome concentration chosen to have the same maximum as membranes) or in buffer (black diamond), for quantification of Hœchst 33342 transport. Data are represented as mean values +/- SEM of an experimental triplicate. **H/** Ratio of Hœchst 33342 transported per ATP hydrolyzed, for each ATP concentration investigated. Each point corresponds to the biological replicate measured in C-F.

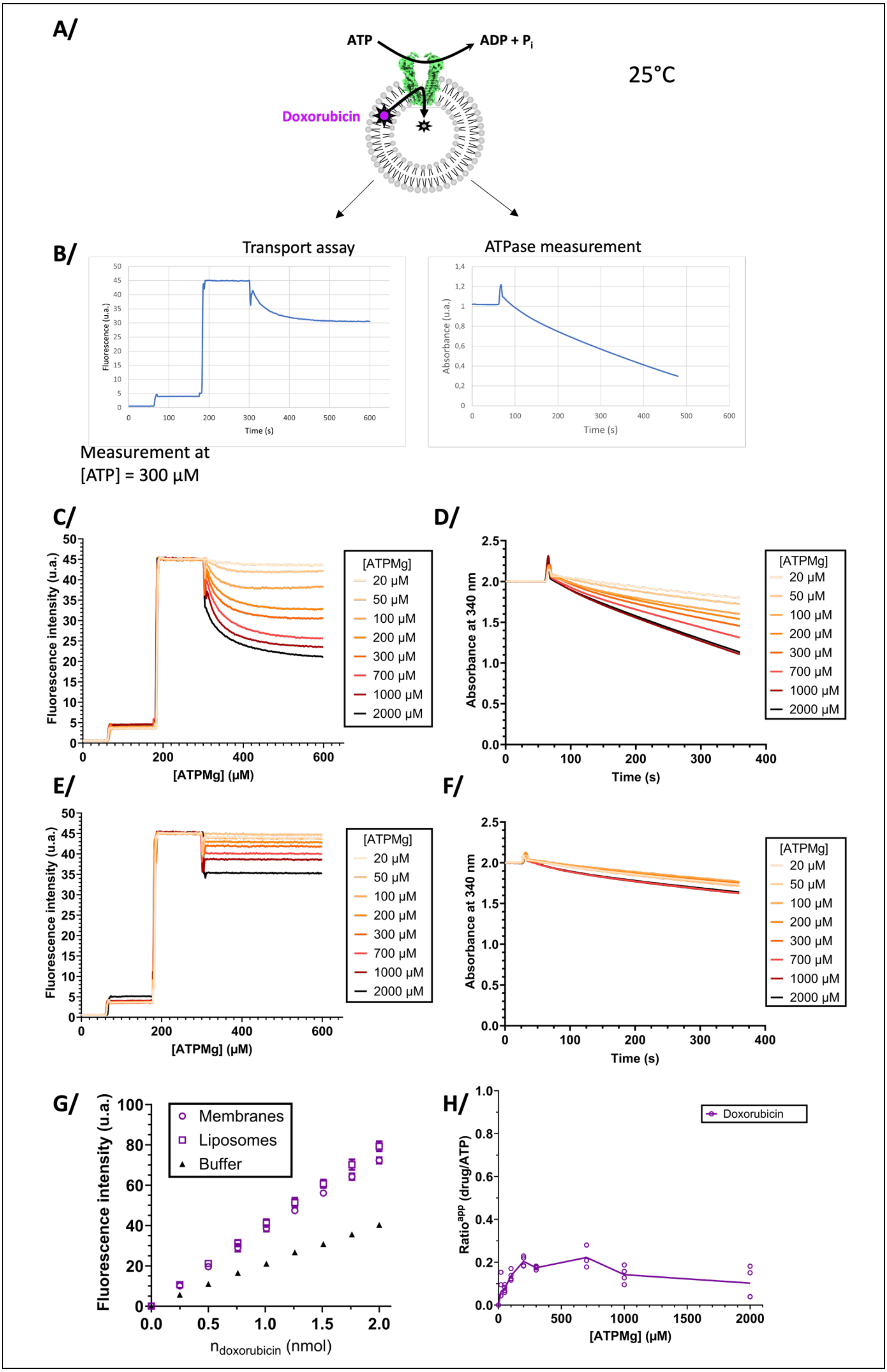

A/
ATP
ADP + Pi
Doxorubicin
25°C
B/
Transport assay
ATPase measurement
Fluorescence (u.a.)
Time (s)
Absorbance (u.a.)
Measurement at
[ATP] = 300 µM
C/
Fluorescence intensity (u.a.)
[ATPMg] (µM)
[ATPMg]
20 µM
50 µM
100 µM
200 µM
300 µM
700 µM
1000 µM
2000 µM
D/
Absorbance at 340 nm
Time (s)
E/
F/
G/
Fluorescence intensity (u.a.)
Membranes
Liposomes
Buffer
n doxorubicin (nmol)
H/
Ratio app (drug/ATP)
Doxorubicin
[ATPMg] (µM)

Supp-Figure 30: Doxorubicin transport. **A/** Schematic representation of the experiment. **B/** Representative traces of substrate transport (left) and ATPase activity (right) for BmrA WT performed on the same membrane vesicles, the same day. All experiments have been carried out in quadruplicates, from 2 different membrane batches. The time range of measurement for transport is shown as a black rectangle on each graph. **C/** One monoplicate of substrate transport for the whole range of ATP tested. **D/** corresponding ATPase activities on the same membranes. C/ and D/ have been measured in quadruplicate available in source data, being 4 biological replicates. **E/ F/** same as C/ and D/ but for the inactive mutant E504A. contaminant activities measured with the mutant E504A were deduced to BmrA WT activities for both transport and ATPase activities. **G/** Standard curve of Doxorubicin fluorescence increase as a function of Doxorubicin being added to a fixed amount of lipid, done on membranes (green circles), liposomes (green squares, liposome concentration chosen to have the same maximum as membranes) or in buffer (black diamond), for quantification of Doxorubicin transport. Data are represented as mean values +/- SEM of an experimental triplicate. **H/** Ratio of Doxorubicin transported per ATP hydrolyzed, for each ATP concentration investigated. Each point corresponds to the biological replicate measured in C-F.

Supplementary Table 1:

Data for E504A without transported substrate.

	**$E504A^{apo}$**	**$E504A^{apo\text{-}25\mu MATP}$**		**$E504A^{apo\text{-}100\mu MATP}$**
**Conformation**	IF	IF	OF	OF
**[ATP-Mg] (µM)**	0	25	25	100
**[R6G] (µM)**	0	0	0	0
**PDB code /**	**8REZ**	**8RGA**	**8RIA**	**8RI1**
**EMD code**	EMD-19113	EMD-19131	EMD-19131	EMD-19180
**Data collection and processing**				
**Magnification**	105,000	105,000	105,000	36,000
**Voltage (kV)**	300	300	300	200
**Electron exposure (e–/Å²)**	40.12	40.12	40.12	43
**Defocus range (µm)**	-1 to -2.4	-1 to -2.4	-1 to -2.4	-1 to -2.4
**Pixel size (Å)**	0.839	0.839	0.839	1.145
**Symmetry imposed**	C2	C2	/	C1
**Initial particle images (no.) (from *ab-initio* job)**	1,005,174	525,711	/	189,344
**Final particle images (no.)**	839,516	525,711	/	189,344<br>Sym. exp.: 378,688
**Map resolution (Å)**	3.3	3.7	/	3,6
**FSC threshold**	0.143	0.143		0.143
**Map resolution range (Å)**	2.8 to 10.7	3.3 to 9.6	/	3.2 to 60.6
**Refinement**				
**Initial model used (PDB code)**	$E504A^{R6G\text{-}25\mu MATP}$	$E504A^{apo}$	/	7BG4
**Model resolution (Å)**	7.7	4.3	/	3.7
**FSC threshold**	0.5	0.5		0,5
**Map sharpening *B* factors (Å²)**	194.6	230.3	/	174,5
**Model composition**			/	
**Non-hydrogen**	8802	8802		8970
**atoms**	1144	1144		1140
**Protein residues**	0	0		4
**Ligands**				
***B* factors (Å²)**			/	
**Protein**	49.86	133.26		109.5
**Ligand**				113.75
**R.m.s. deviations**			/	
**Bond lengths (Å)**	0.017	0.015		0.012
**Bond angles (°)**	3.397	3.151		1.589
**Validation**			/	
**MolProbity score**	3.44	3.11		2.55
**Clashscore**	79.15	61.13		43.69
**Poor rotamers (%)**	3.53	2.39		0.42
**Ramachandran plot**			/	
**Favored (%)**	85.18	89.39		93.4
**Allowed (%)**	13.86	10.09		6.34
**Disallowed (%)**	0.96	0.53		0.26

Data for E504 with transported substrate:

	$E504A^{R6G}$	$E504A^{R6G\text{-}25\mu MATP}$		$E504A^{R6G\text{-}70\mu MATP}$		$E504A^{H33342}$
**Conformation**	IF	IF	OF	OF	IF	IF
**[ATP-Mg] (µM)**	0	25	25	70	70	0
**[R6G] (µM)**	100 µM	100 µM	100 µM	100 µM	100 µM	100
**PDB code /**	**8RF1**	**8RG7**	/	**8RGN**	/	**9GSJ**
**EMD code**	EMD-19115	EMD-19130		EMD-19135		EMD-51550
**Data collection and processing**						
**Magnification**	130,000	130,000	130,000	130,000	130,000	105,000
**Voltage (kV)**	300	300	300	300	300	300
**Electron exposure (e–/Å²)**	40.4	38.8	38.8	37.95	38.8	49.6
**Defocus range (µm)**	-0.8 to -2.2	-1.2 to -3.2	-1.2 to -3.2	-1 to -2.4	-1 to -2.4	-1 to -2.4
**Pixel size (Å)**	1.06	1.052	1.052	1.052	1.052	0.839
**Symmetry imposed**	C1	C2	C2	C2	C1	C1
**Initial particle images (no.) (from *ab-initio* job)**	444,348	625,434	143,956	177,898	170,711	668,794
**Final particle images (no.)**	337,937<br>Sym. exp.: 675,874	150,715	89,293	123,402	288,152	445,152<br>Sym. Exp.: 890,304
**Map resolution (Å)**	4.3	3.9	6.1	3.7	6.2	3.6
**FSC threshold**	0.143	0.143	0.143	0.143	0.143	0.143
**Map resolution range (Å)**	3.6 to 10.7	3.4 to 61.3	5.7 to 15.9	3.2 to 61.5	5.2 to 12.3	3.1 to 8.0
**Refinement**						
**Initial model used (PDB code)**	$E504A^{R6G\text{-}25\mu MATP}$	6R81	/	6R72	/	$E504A^{apo}$
**Model resolution (Å)**	4.3	4.2	/	4.2	/	3.91
**FSC threshold**	0.5	0.5		0.5		0.5
**Map sharpening *B* factors (Å²)**	259.9	185.9	/	181.8	/	198.9
**Model composition**						
**Non-hydrogen**	8868	8868	/	9031	/	8870
**atoms**	1144	1144		1156		1144
**Protein residues**	2	2		6		2
**Ligands**						
***B* factors (Å²)**						
**Protein**	141.8	143.91	/	71.09	/	137.32
**Ligand**	138.69	127.01		71.45		72.86
**R.m.s. deviations**						
**Bond lengths (Å)**	0.009	0.005	/	0.008	/	0.003
**Bond angles (°)**	1.296	0.971		1.351		0.712
**Validation**						
**MolProbity score**	2.07	2.24	/	2.34	/	2.45
**Clashscore**	13.89	16.32	/	30.63		13.17
**Poor rotamers (%)**	0.00	0.52		0.72		3.01
**Ramachandran plot**						
**Favored (%)**	93.6	90.96	/	94.62	/	92.81
**Allowed (%)**	6.4	8.60		4.6		6.93
**Disallowed (%)**	0	0.44		0.78		0.26

Supp-Table 2: MD simulation composition

	**E504A^{apo}**			**E504A^{R6G}**		
**Box composition**						
Protein	1144 residues			1144 residues		
ATP	2			2		
Ligand (RHQ)	-			2		
Mg^{2+} / Na^{+} / Cl^{-}	2 / 180 / 172			2 / 177 / 200		
Lipids*	258 DOPE / 129 DOPG			232 DOPE / 115 DOPG		
Water molecules	66.049			65.099		
Total number of atoms	268.405			264.329		
**Box size**	Replicate 1	Replicate 2	Replicate 3	Replicate 1	Replicate 2	Replicate 3
Before equilibration (Å)	142.72; 140.83; 173.05			143.61; 140.63; 162.50		
After equilibration / start of production (Å)	140.65; 131.85; 161.98	141.90; 135.34; 161.98	139.10; 131.25; 162.80	140.83; 134.03; 152.24	142.34; 135.22; 152.29	143.75; 134.71; 151.63
End of production (Å)	136.82; 136.73; 161.65	137.91; 135.99; 161.642	143.48; 138.38; 162.542	143.78; 140.80; 151.92	144.82; 140.26; 151.97	145.67; 137.36; 151.28

* DOPE: 1,2-dioleoyl-sn- glycero-3-phosphoethanolamine. DOPG: 1,2-dioleoyl-sn-glycero-3-phospho-rac-1-glycerol
