# Supplementary material for: Rhodamine6G and Hœchst33342 narrow BmrA conformational spectrum for a more efficient use of ATP
Source: Nat Commun. 2025 Feb 18;16:1745. doi: 10.1038/s41467-025-56849-z (PMC11836358; doi:10.1038/s41467-025-56849-z)
Supplement: Supplementary file 2 — Description of Additional Supplementary Files [file 41467_2025_56849_MOESM2_ESM.pdf]

Description of Additional Supplementary Files

Supplementary Movie 1: BmrA E504A apo seen from under the NBD, for component 0. Movie loops the 3DVA movement back and forth about 4 times.

Supplementary Movie 2: BmrA E504A apo seen from the membrane plane, for component 0. Movie loops the 3DVA movement back and forth about 4 times.

Supplementary Movie 3: BmrA E504A apo seen from under the NBD, for component 1. Movie loops the 3DVA movement back and forth about 4 times.

Supplementary Movie 4: BmrA E504A apo seen from the membrane plane, for component 1. Movie loops the 3DVA movement back and forth about 4 times.

Supplementary Movie 5: BmrA E504A apo seen from under the NBD, for component 2. Movie loops the 3DVA movement back and forth about 4 times.

Supplementary Movie 6: BmrA E504A apo seen from the membrane plane, for component 2. Movie loops the 3DVA movement back and forth about 4 times.

Supplementary Movie 7: BmrA E504A-R6Gseen from under the NBD, for component 0. Movie loops the 3DVA movement back and forth about 4 times.

Supplementary Movie 8: BmrA E504A-R6G seen from the membrane plane, for component 0. Movie loops the 3DVA movement back and forth about 4 times.

Supplementary Movie 9: BmrA E504A-R6G seen from under the NBD, for component 1. Movie loops the 3DVA movement back and forth about 4 times.

Supplementary Movie 10: BmrA E504A-R6G seen from the membrane plane, for component 1. Movie loops the 3DVA movement back and forth about 4 times.

Supplementary Movie 11: BmrA E504A-R6G seen from under the NBD, for component 2. Movie loops the 3DVA movement back and forth about 4 times.

Supplementary Movie 12: BmrA E504A-R6G seen from the membrane plane, for component 2. Movie loops the 3DVA movement back and forth about 4 times.
